# Supplementary material for: Barriers to uptake and use of pre-exposure prophylaxis (PrEP) among communities most affected by HIV in the UK: findings from a qualitative study in Scotland
Source: BMJ Open. 2014 Nov 20;4(11):e005717. doi: 10.1136/bmjopen-2014-005717 (PMC4244494; doi:10.1136/bmjopen-2014-005717)
Supplement: Author's manuscript [file bmjopen-2014-005717.draft_revisions.pdf]

BMJ Open

Barriers to uptake and use of Pre-Exposure Prophylaxis (PrEP) amongst communities most affected by HIV in the UK

Journal:	*BMJ Open*
Manuscript ID:	bmjopen-2014-005717
Article Type:	Research
Date Submitted by the Author:	16-May-2014
Complete List of Authors:	Young, Ingrid; University of Glasgow, MRC/CSO Social and Public Health Sciences Unit<br>Flowers, Paul; Glasgow Caledonian University, School of Health and Life Sciences<br>McDaid, Lisa; University of Glasgow, MRC/CSO Social and Public Health Sciences Unit
**Primary Subject Heading**:	HIV/AIDS
Secondary Subject Heading:	Qualitative research, Public health, Sexual health, Sociology
Keywords:	HIV & AIDS < INFECTIOUS DISEASES, QUALITATIVE RESEARCH, PUBLIC HEALTH

SCHOLARONE™
Manuscripts

For peer review only

Barriers to uptake and use of Pre-Exposure Prophylaxis (PrEP) amongst communities most affected by HIV in the UK

Ingrid Young, Paul Flowers, Lisa McDaid

Running Head: Barriers to Pre-Exposure Prophylaxis (PrEP) in Scotland

Authors: IYoung[1], P Flowers[2], LM McDaid[1]

Key Words: Pre-Exposure Prophylaxis (PrEP), men who have sex with men (MSM), migrant African communities, Scotland, biomedical HIV prevention

Affiliation:

1. MRC/CSO Social and Public Health Sciences Unit, University of Glasgow
2. School of Health and Life Sciences, Glasgow Caledonian University

Corresponding Author:

Ingrid Young,
MRC/CSO Social and Public Health Sciences Unit, University of Glasgow
200 Renfield Street
Glasgow, G2 3QB
0141 353 7533
ingrid.young@glasgow.ac.uk

For peer review only - http://bmjopen.bmj.com/site/about/guidelines.xhtml

Abstract

Objectives: To explore the acceptability of Pre-Exposure Prophylaxis (PrEP) amongst gay, bisexual and men who have sex with men (MSM) and migrant African communities in Scotland, UK.

Design: Consecutive mixed qualitative methods using focus groups and in-depth interviews were conducted to explore the acceptability of PrEP. Data were digitally recorded, transcribed and analysed thematically to identify anticipated and emerging themes

Setting: Participants recruited through community sexual health & outreach support services, and from non-sexual health settings across urban and semi-urban Scotland.

Participants: Inclusion criteria included: identification as either MSM and/or from migrant African communities; 18 years and older; living in Scotland at the time of participation. 7 focus groups were conducted (n=33): 5 with MSM (n=22), and 2 with African participants (n=11, women=8), aged 18 – 75 years. 34 in-depth interviews were conducted with MSM (n=20) and African participants (n=14, women = 10), aged 19 – 60 years. The sample included participants who were HIV-positive and HIV-negative or untested (HIV-positive participants in focus groups, n=22; HIV-positive participants in interviews, n=17).

Results: Perceptions of PrEP effectiveness, HIV literacy and concerns about maintaining regular adherence were identified as barriers to PrEP uptake and use. Low perception of HIV risk as a result of existing risk management strategies meant that few participants saw themselves as PrEP candidates. Participants identified risk of other STIs and pregnancy as a concern which PrEP did not address for either themselves or their sexual partners. PrEP emerged as a contentious issue because of the potentially negative implications it had for HIV prevention. Many participants viewed PrEP as problematic because they perceived that *others* would stop using condoms if PrEP was to become available.

For peer review only - http://bmjopen.bmj.com/site/about/guidelines.xhtml

Conclusions: PrEP implementation will need to address HIV literacy, risk reduction concerns, and how it can be part of a safe and comprehensive risk management strategy.

Article summary/Strengths and limitations of this study

- Our study offers new insights into the psychological and social barriers to PrEP uptake and use.
- This paper highlights the importance of HIV literacy in effective and efficient PrEP uptake and use.
- We demonstrate the need to consider socially embedded sexual risk management strategies, which address concerns beyond risk of HIV transmission.
- We suggest there is a need to consider how clinical research is translated into real world contexts and communicated to potential users, as well as how potential users are supported in integrating this information into existing risk management strategies.

For peer review only

For peer review only - http://bmjopen.bmj.com/site/about/guidelines.xhtml

Pre-Exposure Prophylaxis (PrEP) represents a significant biomedical addition to international HIV prevention efforts. The use of anti-retrovirals (ARVs) by HIV-negative individuals to prevent the sexual transmission of HIV has been widely tested and its clinical efficacy has been extensively reported (1, 2). There are currently a number of PrEP demonstration studies underway in the United Kingdom (UK), Australia, United States (US) and elsewhere to explore how it might be used in 'real world' settings (3). However, the acceptability of PrEP to potential PrEP users (or candidates), has received significantly less attention (4, 5). Most research has focused on the risk of inefficient PrEP use by potential candidates, such as low or inconsistent adherence to the medication and reduced condom use. It has also focused predominately on US experiences. Despite being available in the US since the Food and Drug Administration (FDA) approved Truvada (emtricitabine/tenofovir disoproxil fumarate) for use as PrEP in 2012, there has been relatively low uptake to date amongst targeted communities affected by HIV, including gay, bisexual and other men who have sex with men (MSM) (3). Moreover, PrEP has emerged as a controversial issue amongst those affected by HIV(6) and there are clear global inequalities in terms of access to PrEP as it is not available in a number of countries, including the UK.

There is a need to understand the acceptability of PrEP, how the uptake and use of PrEP as an HIV prevention strategy could be affected by a range of factors, such as the social environment in which it is introduced, and the role of PrEP within concurrent HIV prevention strategies of potential PrEP users (7, 8). Here we describe a qualitative study of the social barriers to PrEP uptake and use amongst populations most affected by HIV in Scotland, UK to inform the implementation of PrEP in low-prevalence settings.

Methods

For peer review only - http://bmjopen.bmj.com/site/about/guidelines.xhtml

We undertook a mixed qualitative methods study of the acceptability of TasP with two communities in Scotland: 1) MSM and 2) men and women from migrant African communities. These communities represent over half of all new HIV diagnoses in Scotland, as well as across the UK (9, 10). We included HIV-negative and/or untested participants as potential PrEP users, and HIV-positive participants as potential or existing sexual partners of PrEP users. The latter group were included because of evidence which indicates that serodiscordant sexual partners may be an important factor in PrEP acceptability (11).

First, exploratory focus groups (FGs) were conducted with a convenience sample of MSM and African men and women between August and November 2012. We conducted seven FGs: five with MSM (n=22), and two with African participants (n=11). Three FGs were conducted with HIV-positive MSM (n=14) and one FG with HIV-positive Africans (n=8). The age range of participants was between 18 and 75 years and discussions took place across urban and semi-urban locations in Scotland (Glasgow, Edinburgh, Motherwell and Selkirk). Participants were recruited through existing community and/or support groups with the assistance of sexual health and/or LGBT organisations. FG discussion topics included existing risk management strategies in sexual health, use of sexual health technologies (e.g. condoms, testing, ARVs, etc) and biomedical HIV prevention interventions (PrEP and Treatment as Prevention). PrEP was explained with the use of visual aids (Figure 1). Material did not specify an efficacy rate, but, when asked, participants were informed that it was dependent on levels of adherence and that the iPrEx study reported approximately 73% protection, which was accurate at the time discussions were conducted (12).

We then conducted 34 in-depth interviews (IDIs) between March and September 2013 with a purposive sample of MSM (n=20) and African men and women (n=14). Half of the IDI

For peer review only - http://bmjopen.bmj.com/site/about/guidelines.xhtml

participants were HIV-negative or untested for HIV, and the other half had been diagnosed with HIV at the time of the interview (MSM, n=10; Africans, n=7). IDI participants were aged 19 –60 years and were resident in four Scottish regions (Glasgow, Lothian, Lanarkshire, and Grampian). Tailored flyers and posters were distributed to: workshops and support groups, including meetings and events for people living with HIV; community HIV and STI testing clinics; gay bars, saunas and clubs; a mail-out condom programme for MSM; commercial venues such as African food and barber shops; and in educational settings known to have large African student populations. Community organisations also made contact with potential participants through their regular online and face-to-face outreach and support work. Interviews took place in locations convenient to participants, including private offices, university classrooms, meeting rooms or in participants' own homes. The first part of the semi-structured interview focused on personal experiences of and perspectives on HIV, sexual health risk management practices, and use of sexual health technologies (as before). The second part of the interview focused on the acceptability of PrEP and TasP, focusing on awareness, potential use, concerns and combination with existing risk management strategies.

.

All FGs and IDIs were digitally recorded and transcribed verbatim. Transcripts were anonymised and coded in NVivo 10. Data were analysed thematically, drawing on both anticipated and emergent themes (13-15). Rigour throughout the analysis was achieved through an iterative process of discussion and revision between co-authors (13, 14, 16). We have identified extracts taken from FGs; otherwise, it can be assumed that the extract comes from an IDI participant. Ethical approval was obtained from the University of Glasgow's College of Social Sciences Ethics Committee (CSS2012/0193, CSS20120264).

For peer review only - http://bmjopen.bmj.com/site/about/guidelines.xhtml

Results

We identified five potential barriers to effective PrEP uptake and use: interpreting effectiveness and HIV literacy; managing adherence to PrEP; PrEP candidacy and low perceptions of HIV risk; concerns with other risks such as the criminalization of HIV transmission and STIs; and moral barriers.

Interpreting effectiveness and HIV literacy

Perceptions of PrEP effectiveness emerged as a significant barrier to potential use. Most participants expressed concerns about its effectiveness to prevent HIV transmission. These concerns were often grounded in the less than 100% efficacy rates and a perception that PrEP provided 'insufficient' protection from HIV transmission on its own. Some participants felt that PrEP used in isolation was 'too much' of a risk (if only approximately 70% effective in clinical trials).

> ***R1****: That's not enough, that's not enough, exactly.*
>
> ***R2:*** *Its seventy-two per cent effective but then there's still that twenty-eight per cent.*
>
> (HIV-Negative MSM, FG)

Participants also expressed a wider scepticism with regards to PrEP, which emerged in two ways. Some thought that the advice to use condoms with PrEP indicated a continued scientific uncertainty: '*I don't know if this [will] work because since they said if you use it you can still use condom, that means they are not sure as well'* (HIV-negative African woman). Second, some expressed scepticism in relation to the variability in reported efficacy rates as described in relation to adherence. As a result, there was a reluctance to trust these, unless the information came from a recognised, trusted and reliable source:

For peer review only - http://bmjopen.bmj.com/site/about/guidelines.xhtml

R1: *There's so much out there right, on the internet an' everything else right, if it came up on the news right? The logistic news on BBC one that this pill will prevent so…—like a hundred per cent – that's when I would believe it would work. I [would not] trust any other pill ...*

Q: *So it has to be a hundred per cent?*

R2: *'cause there's a lot a... no like—anyone can put something on the internet,... You've got to use like certain websites, you've got to look like .org, .gov, those type o' websites. You've just done one that's like www.medicine.co.uk it's not a... it's not like a legitimate website it could just be anyone postin' stuff up there.*

Q: *Well I suppose in the States it's from the Food and Drug Administration so it's got that stamp on it.*

R1*: Yes, but you [don't know] if they're been a hundred per cent accurate on their job or just been lazy an' the fact that we're brave enough to take it to human trials right?*

(HIV-Negative MSM, FG)

In spite of being told that the FDA had approved the drug, these FG participants did not know the organisation or trust it as a reliable source, and were therefore sceptical about its statements and/or endorsement.

Other participants were less concerned about PrEP not being 100% efficacious because they imagined that it would be used in addition to condoms. However, participants expressed confusion or uncertainty in knowing how to interpret efficacy rates and their impact on risk reduction in this particular context. For example, in discussing the effectiveness of condoms in relation to other prevention strategies, one participant added up the protection offered by

For peer review only - http://bmjopen.bmj.com/site/about/guidelines.xhtml

PrEP, condoms and TasP with a hypothetical HIV-positive sexual partner. Assuming that PrEP was 90% effective at reducing HIV transmission, he explained:

> ***R***: *but there's obviously still a 10% risk but, as you said, there's the same risk with condoms. So it's either you take the 10% risk or you say 'well, I'll use condoms and we'll use TASP' which makes 180%. (Laugh)*
>
> ***Q***: *And PrEP. (Laugh)*
>
> ***R***: *253 out of 300!*
>
> (HIV-negative MSM)

Although the participant in this extract was joking about adding up the numbers, the interpretation of efficacy rates in relation to other prevention options posed a potential source of confusion or misinformation for participants.

Managing adherence to PrEP

Given the evidence concerning the patterning of efficacy by adherence (2), maintaining regular adherence to medication was identified as a potential barrier to effective PrEP use. Some participants described how they might forget to take tablets or their routine might be disrupted because of non-regular working patterns, and were therefore concerned about how effective PrEP would be if they did not take the drugs regularly: '*Sometimes I forget. Because I don't have a regular sleeping pattern, so sometimes I'll fall asleep at four in the afternoon and I usually take my pills [for an existing health condition] after dinner'* (HIV-Negative MSM). Establishing and maintaining a routine to take PrEP was also affected by the perception of social stigma attached to HIV medication. This meant maintaining daily adherence would be difficult if there was limited privacy (e.g. the presence of room-mates) or if there was a change in environment. One participant reported concerns about his family potentially finding the tablets:

For peer review only - http://bmjopen.bmj.com/site/about/guidelines.xhtml

...if I was on those medications for a year and if I went home...or anything like that I'd find it very difficult to be able to take my medications or I would find it a bit of a barrier that if my family knew about it they'd investigate why are you on these pills. And again that would probably put some doubt in their head and then they'd probably then think the worst - that I was HIV positive.

(HIV-negative MSM)

In addition to being unable to establish or maintain a regular routine to facilitate good adherence, most participants expressed concerns about the physical effects of the drugs themselves, and how the side-effects might inhibit taking them. Some participants described disliking taking tablets and reported that this would be a barrier to daily PrEP use: *I don't really like pills so I don't think I would take any pills every day'* (HIV-negative African man). Many viewed the potential for side effects, such as nausea and diarrhoea, as too great a trade-off for increased HIV prevention, in spite of being informed that trials had reported high tolerance of the drugs:

But, could I put up wi' side-effects all the time as well, like taking a pill, and going tae a nightclub, and ended up wi' diarrhoea? You know, you've gotta think of things like that, am I gonna take this? Am I gonnae stand tired, have a headache in a club just at the fact that I might at the end of it get a shag...(HIV-Negative MSM)

Other participants felt the side-effects would interfere with sexual practice itself: '*Nausea, an' diarrhoea an'—you'll be shittin' all over his knob. No ta.'* (HIV-negative MSM, FG) A significant minority of both HIV-positive and HIV-negative participants raised the issue of longer-term side-effects, especially those who were more familiar with the effects of ARVs:

Does it not have any side effects inside my body? 'Cause from my reading as well, some of these anti-retrovirals have got side effects with the lungs, with the kidneys

For peer review only - http://bmjopen.bmj.com/site/about/guidelines.xhtml

For peer review only

> *and stuff. So, in the long run, if I get used to drinking this pill and I'm actually not exposed because I'm thinking I might be at risk of getting exposed, am I not doing more damage to myself in my body?* (HIV-Negative African woman)

Many participants described scepticism in taking PrEP daily, especially if they were not always exposed to or at risk of HIV transmission.

PrEP candidacy and low perceptions of HIV risk

Perceptions of HIV transmission risk played an important role in potential uptake of PrEP. Several participants rejected the use of PrEP because they perceived themselves to be at a very low risk. For the majority of HIV-negative participants, this was because most could not imagine knowingly having an HIV-positive sexual partner: '*I wouldn't just wanna take a risk because it's already defining as a risk, I mean, it's a risk, it's already been defined as a risk and since I'm a little bit risk averse... I won't just do anything'* (HIV-negative African man). Some HIV-negative participants assumed that HIV-disclosure to sexual partners '*was the law'* (HIV-negative MSM, FG), and that their sexual partners were not HIV-positive. Those HIV-negative participants in sero-discordant relationships and HIV-positive participants suggested that condoms, and the nature of their relationships (e.g. monogamous), already helped manage their risks, and therefore, they did not think that the additional benefit from PrEP was necessary. One man in a sero-discordant relationship explained:

> *Right now in my current situation as, I'm doing a monogamous relationship and everything like that, again I don't think even in that [instance] I don't think I would take the pills even though it would be an extra measure...I think I would feel comfortable enough in the current situation.* (HIV-Negative MSM)

For peer review only - http://bmjopen.bmj.com/site/about/guidelines.xhtml

For peer review only

PrEP and concerns with other risks

Both HIV-positive and HIV-negative participants identified risk of other sexually-transmitted infections (STIs) as a concern for both themselves and their sexual partners. Heterosexual African participants, especially African women, also talked of the risk of pregnancy. For those participants who were either living with HIV, or who had experience of sero-discordant relationships, other health risks posed to the HIV-positive sexual partner through PrEP use was also identified:

> *when I'm looking at condom use it's not just HIV that you're protecting yourself against but some other STIs as well. So you're killing two birds with one stone, more like it. But when it's now using this, you're actually getting yourself involved in something that you already know – 'this person is positive and I'm gonna have sex with him, but it's okay, I will not use condoms because I know I can use this.' But you're also putting that person, because that person's immune system might be weak. They're also putting that person at risk of other STIs as well, instead of using condoms. So I think it makes people more ignorant of the other things as well. And it makes people just more focused on just HIV and not other STIs. And then risk of pregnancy as well is likely to get high, provided the people are not on any other form of contraception.... No, I think... no, from the way you've said it to me... I wouldn't even tell it to people* (HIV-negative African woman)

Most HIV-positive participants described anxieties about PrEP in relation to *their* inability to be in effective control of HIV prevention. One woman could not imagine agreeing to her HIV-negative sexual partner using PrEP: *'[PrEP] is too risky for him because I don't know when he stop using it, what will happen to him.'* (HIV-positive African woman, FG).

For peer review only - http://bmjopen.bmj.com/site/about/guidelines.xhtml

Moral barriers to PrEP

PrEP also emerged as a contentious issue because of the perceived negative implications it had for existing risk management strategies and HIV prevention. Many participants viewed PrEP as problematic because they perceived that *others* would stop using condoms if PrEP was available. That is, they distinguished between how *they* might use PrEP and how they perceived others would use it. For some, the concern was in relation to reduced condom use: '*it'll be like... women burning their bras. It'll be all these guys [whipping] off their condoms, do you know what I mean?'* (HIV-negative MSM). Other participants were also concerned with bigger changes in sexual practice, such as increased risk taking:

> *Are you trying to eliminate the condom?... There is no place for a condom at all when it's like this. So I think it actually encourages people to be more promisc... not promiscuous, but to be more... I don't know [if] 'ignorant' is the right word, but to be less careful 'cause they know 'oh, there's that pill'. And it's not even cost-effective than just getting a condom or using...PEP once in a blue moon.* (HIV-negative African woman)

Not all participants were morally opposed to PrEP and many viewed PrEP as a good addition to HIV prevention options. However, there was still concern about *how* PrEP would be accessed and overall it was hoped that it would be made available only under strict conditions to ensure the 'right' people received it and that it was used in the 'correct' way:

> *I believe it should be available in Scotland. But, it should be under strict control to make sure the person who takes it knows that he, or she is not protected to full extent from other infections, and HIV either.* (HIV-negative MSM)

For peer review only - http://bmjopen.bmj.com/site/about/guidelines.xhtml

Discussion

This is the first qualitative study in the UK to report on the acceptability of PrEP amongst potential PrEP users and their HIV-positive sexual partners. Our study offers new insights into the psychological and social barriers to PrEP uptake and use. We identified the importance of HIV literacy, as well as related HIV risk perception and found that, for many participants, PrEP was not immediately seen as a trusted and/or beneficial addition to their repertoire of existing risk reduction practices. Our findings also highlight how existing risk management strategies are about more than HIV risk, and encompass broader concerns relating to sexual health, relationships, social factors and communities..

Understanding how to interpret PrEP efficacy rates, both on their own and in combination with other prevention strategies, proved a stumbling block for the participants. Although Liu *et al* identify accurate consumer knowledge as a key to PrEP implementation (17), our findings suggest that *more* than consumer knowledge is needed for informed and effective PrEP use. Negotiating PrEP as a prevention strategy, either on its own and with other strategies, will require improved levels of HIV literacy to be effective (we define HIV literacy as the ability to know, understand and potentially utilise HIV-related information within existing risk reduction practices). Although we did not sample or analyse the data by educational qualification or socio-economic status, our findings suggest that social inequalities (e.g sexuality, geography, migration status, etc), directly affect HIV literacy, and will play an important role in understanding the barriers to PrEP uptake amongst diverse communities affected by HIV. These factors will have direct implications for both the nature of how PrEP-related HIV risk prevention is delivered and by whom.

For peer review only - http://bmjopen.bmj.com/site/about/guidelines.xhtml

For many participants, PrEP was not seen as a necessary or welcome addition to their repertoire of risk management strategies. As findings from the VOICE trial attest to, risk perception and candidacy play a critical role in the successful use of PrEP (18). For some of the HIV-negative participants in our study, the rejection of PrEP as unnecessary emerged from a perception that they were not at risk of HIV transmission. HIV risk was managed at a different level, often through the sexual exclusion of HIV-positive sexual partners or through reliance on monogamous sexual relationships. These findings suggest that PrEP implementation strategies will need to engage with these wider, socially embedded risk reduction practices. Moreover, our research demonstrates how the management of risk was not limited only to HIV transmission, but also to risk of STIs, pregnancy and social stigma. Participants described how PrEP would not adequately address existing risks, and had the potential to create significant new risks. Saberi *et al* reported similar moral concerns about the implications of PrEP on condom use in their study with MSM participants in serodiscordant relationships in the US (19). Although they surmise that proximity to HIV and age play a role in these concerns, our findings suggest that moral objections to PrEP were not limited by age or sexuality. This highlights the need to engage with wider social concerns about inappropriate or high-risk sexual practice in relation to PrEP and to demonstrate how PrEP implementation can be a part of a safe and comprehensive risk management strategy.

Our study has a number of strengths and limitations. We employed well-developed and rigorous qualitative methodology which enables in-depth exploration of the social meanings of PrEP acceptability and likely use (15). As such, this study adds to existing quantitative PrEP acceptability research. With a small sample of MSM and migrant African participants from a low HIV prevalence setting, some of whom were engaged in sexual health or

For peer review only - http://bmjopen.bmj.com/site/about/guidelines.xhtml

community services, our findings are not generalisable to a wider population but we would argue, are transferable to similar populations in similar social contexts.

Our research demonstrates the need to consider acceptability factors which extend beyond drug adherence and risk compensation when introducing and scaling up PrEP and has a number of implications for policy and clinical practice. In particular, it will be necessary to consider how clinical research concerning effectiveness is translated into real world contexts and communicated to potential users, as well as how potential users are supported in integrating this information into existing risk management strategies. Further research is needed to examine HIV literacy in relation to social inequalities in the understanding of complex trial-based information explicating probabilistic and relative risk. For the effective and efficient implementation of PrEP as a viable prevention strategy, future work is needed to develop clear tools and techniques to communicate PrEP information to potential candidates and to explore the need for the targeted promotion of PrEP.

Competing Interests

We have no competing interests.

Author's Contributions

IY designed the study, carried out the qualitative data collection and analysis and drafted the manuscript. PF helped to design the study, carried out some of the data collection and analysis and helped to draft the manuscript. LMD participated in the design of the study and helped to draft the manuscript. All authors read and approved the final manuscript.

Acknowledgements

For peer review only - http://bmjopen.bmj.com/site/about/guidelines.xhtml

The HIV and the Biomedical Study, IY and LMD are core funded by the UK Medical Research Council (MRC) (MC_U130031238/MC_UU_12017/2) at the MRC/Chief Scientist Office (CSO) Social and Public Health Sciences Unit, University of Glasgow. PF is funded by Glasgow Caledonian University. We thank the organisations who helped with recruitment and the men and women who agreed to take part in the focus groups and interviews.

References

1. Baeten JM, Grant R. Use of Antiretrovirals for HIV Prevention: What Do We Know and What Don't We Know? Current HIV/AIDS Reports. 2013;10(2):142-51.

2. Anderson PL, Glidden DV, Liu A, Buchbinder S, Lama JR, Guanira JV, et al. Emtricitabine-Tenofovir Concentrations and Pre-Exposure Prophylaxis Efficacy in Men Who Have Sex with Men. Science Translational Medicine. 2012;4(151):1-8.

3. Kirby T, Thornber-Dunwell M. Uptake of PrEP for HIV slow among MSM. Lancet. 2014;383(9915):399-400.

4. Young I, McDaid L. How Acceptable are Antiretrovirals for the Prevention of Sexually Transmitted HIV?: A Review of Research on the Acceptability of Oral Pre-exposure Prophylaxis and Treatment as Prevention. AIDS Behav. 2014 Jul 30;18 (2):195-216(2):195-216.

5. Holt M. HIV pre-exposure prophylaxis and treatment as prevention: a review of awareness and acceptability among men who have sex with men in the Asia-Pacific region and the Americas. Sex Health. 2013 Jul 19.

6. Cairns G. The road to PrEP: trials, regulation and roll-out. aidsmap. 2012.

7. Kippax S, Stephenson N. Beyond the distinction between biomedical and social dimensions of HIV prevention through the lens of a social public health. Am J Public Health. 2012 May;102(5):789-99.

For peer review only - http://bmjopen.bmj.com/site/about/guidelines.xhtml

8. Nguyen VK, Bajos N, Dubois-Arber F, O'Malley J, Pirkle CM. Remedicalizing an epidemic: from HIV treatment as prevention to HIV treatment is prevention. AIDS. 2011 Jan 28;25(3):291-3.

9. Health Protection Scotland. ANSWER: HIV infections and AIDS in Scotland: Quarterly report to 30 June 2013: Health Protection Scotland2013 4 September 2013 Contract No.: 2013/36.

10. PHE. HIV in the United Kingdom: 2013 Report. London: Public Health England2013.

11. Ware NC, Wyatt MA, Haberer JE, Baeten JM, Kintu A, Psaros C, et al. What's love got to do with it? Explaining adherence to oral antiretroviral pre-exposure prophylaxis for HIV-serodiscordant couples. J Acquir Immune Defic Syndr. 2012 Apr 15;59(5):463-8.

12. Baeten J, Celum C. Oral antiretroviral chemoprophylaxis: current status. Curr Opin HIV AIDS. 2012 Sep 7;7(7):514-9.

13. Mason J. Qualitative Researching. London: Sage Publications; 1996.

14. Barbour R, Kitzinger J, editors. Developing Focus Group Research: Politics, Theory and Practice. London: Sage Publications; 1999.

15. Ziebland S, McPherson A. Making sense of qualitative data analysis: an introduction with illustrations from DIPEx (personal experiences of health and illness). Medical Education. 2006;40(5):405-14.

16. Silverman D. Doing Qualitative Research: A Practical Handbook. London: Sage; 2000.

17. Liu A, Cohen S, Follansbee S, Cohan D, Weber S, Sachdev D, et al. Early Experiences Implementing Pre-exposure Prophylaxis (PrEP) for HIV Prevention in San Francisco. PLoS Med. 2014;11(3):e1001613.

For peer review only - http://bmjopen.bmj.com/site/about/guidelines.xhtml

For peer review only

18. van der Straten A, Stadler J, Montgomery E, Hartmann M, Magazi B, Mathebula F, et al. Women's Experiences with Oral and Vaginal Pre-Exposure Prophylaxis: The VOICE-C Qualitative Study in Johannesburg, South Africa. PLoS ONE. 2014;9(2):e89118.

19. Saberi P, Gamarel KE, Neilands TB, Comfort M, Sheon N, Darbes LA, et al. Ambiguity, ambivalence, and apprehensions of taking HIV-1 pre-exposure prophylaxis among male couples in San Francisco: a mixed methods study. PLoS One. 2012;7(11):e50061.

For peer review only

For peer review only - http://bmjopen.bmj.com/site/about/guidelines.xhtml

Figure 1.

PrEP (Pre-Exposure Prophylaxis)

**Who is it for?**	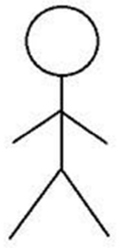 **Person <u>not living with HIV</u> (HIV negative)**
**What is it?**	ARVs are taken **before exposure to HIV** (usually before having sex with someone living with HIV)
**What does this involve?**	PrEP would mostly likely be a pill that would be taken every day. Side effects could include nausea, diarrhoea, headaches, and tiredness.
**Does this work?**	PrEP is not 100% effective, but **when used with condoms, significantly reduces risk of HIV transmission**.
**Can I get it here?**	No, PrEP is not currently available in Scotland or anywhere else in the UK.

BMJ Open

Barriers to uptake and use of Pre-Exposure Prophylaxis (PrEP) amongst communities most affected by HIV in the UK: Findings from a qualitative study in Scotland

Journal:	*BMJ Open*
Manuscript ID:	bmjopen-2014-005717.R1
Article Type:	Research
Date Submitted by the Author:	16-Sep-2014
Complete List of Authors:	Young, Ingrid; University of Glasgow, MRC/CSO Social and Public Health Sciences Unit<br>Flowers, Paul; Glasgow Caledonian University, School of Health and Life Sciences<br>McDaid, Lisa; University of Glasgow, MRC/CSO Social and Public Health Sciences Unit
**Primary Subject Heading**:	HIV/AIDS
Secondary Subject Heading:	Qualitative research, Public health, Sexual health, Sociology
Keywords:	HIV & AIDS < INFECTIOUS DISEASES, QUALITATIVE RESEARCH, PUBLIC HEALTH

SCHOLARONE™
Manuscripts

For peer review only

For peer review only - http://bmjopen.bmj.com/site/about/guidelines.xhtml

Barriers to uptake and use of Pre-Exposure Prophylaxis (PrEP) amongst communities most affected by HIV in the UK: Findings from a qualitative study in Scotland

Ingrid Young, Paul Flowers, Lisa McDaid

Running Head: Barriers to Pre-Exposure Prophylaxis (PrEP) in the UK

Authors: IYoung[1], P Flowers[2], LM McDaid[1]

Key Words: Pre-Exposure Prophylaxis (PrEP), men who have sex with men (MSM), migrant African communities, Scotland, biomedical HIV prevention

Affiliation:

1. MRC/CSO Social and Public Health Sciences Unit, University of Glasgow
2. School of Health and Life Sciences, Glasgow Caledonian University

Corresponding Author:

Ingrid Young,
MRC/CSO Social and Public Health Sciences Unit, University of Glasgow
200 Renfield Street
Glasgow, G2 3QB
0141 353 7533
ingrid.young@glasgow.ac.uk

For peer review only - http://bmjopen.bmj.com/site/about/guidelines.xhtml

For peer review only

Abstract

Objectives: To explore the acceptability of Pre-Exposure Prophylaxis (PrEP) amongst gay, bisexual and men who have sex with men (MSM) and migrant African communities in Scotland, UK.

Design: Consecutive mixed qualitative methods consisting of focus groups (FGs) and in-depth interviews (IDI) explored PrEP acceptability. Data were digitally recorded, transcribed and analysed thematically to identify anticipated and emerging themes.

Setting: Participants were recruited through community sexual health and outreach support services, and from non-sexual health settings across Scotland.

Participants: Inclusion criteria included: identification as either MSM and/or from migrant African communities; 18 years and older; living in Scotland at the time of participation. 7 FGs were conducted (n=33): 5 with MSM (n=22), and 2 mixed-sex groups with African participants (n=11, women=8), aged 18 – 75 years. 34 IDIs were conducted with MSM (n=20) and African participants (n=14, women = 10), aged 19 – 60 years. The sample included participants who were HIV-positive and HIV-negative or untested (HIV-positive FG participants, n=22; HIV-positive IDI participants, n=17).

Results: Understandings of PrEP effectiveness and concerns about maintaining regular adherence were identified as barriers to PrEP uptake and use. Low perception of HIV risk due to existing risk management strategies meant few participants saw themselves as PrEP candidates. Participants identified risk of other STIs and pregnancy as a concern which PrEP did not address for either themselves or their sexual partners. PrEP emerged as a contentious issue because of the potentially negative implications it had for HIV prevention. Many participants viewed PrEP as problematic because they perceived that *others* would stop using condoms if PrEP was to become available.

For peer review only - http://bmjopen.bmj.com/site/about/guidelines.xhtml

Conclusions: PrEP implementation needs to: identify appropriate communication methods in the context of diverse HIV literacy; address risk reduction concerns and; demonstrate how PrEP can be part of a safe and comprehensive risk management strategy.

Article summary/Strengths and limitations of this study

- Our study offers new insights into the psychological and social barriers to PrEP uptake and use.
- This paper identifies how inequalities in critical HIV literacy and wider community attitudes affect PrEP acceptability.
- We demonstrate the need to consider socially embedded sexual risk management strategies, which address concerns beyond risk of HIV transmission.
- We suggest there is a need to consider how clinical research is translated into real world contexts and communicated to potential users, as well as how potential users are supported in integrating this information into existing risk management strategies.

For peer review only

For peer review only - http://bmjopen.bmj.com/site/about/guidelines.xhtml

Pre-Exposure Prophylaxis (PrEP) represents a significant biomedical addition to international HIV prevention efforts. The clinical efficacy of the use of anti-retrovirals (ARVs) by HIV-negative individuals to prevent the sexual transmission of HIV has been extensively reported (1, 2). The acceptability of PrEP to potential PrEP users (or candidates) to date has focused largely, although not exclusively, on the risk of inefficient PrEP use, such as low or inconsistent adherence to the medication and reduced condom use. It has also focused predominately on experiences in the United States (US) (3-5). There are currently a number of PrEP demonstration studies underway in the United Kingdom (UK), Australia, US and elsewhere to explore how it might be used in 'real world' settings (6, 7). Despite being available in the US since the Food and Drug Administration (FDA) approved Truvada (emtricitabine/tenofovir disoproxil fumarate) for use as PrEP in 2012, there has been relatively low uptake amongst targeted communities affected by HIV, including gay, bisexual and other men who have sex with men (MSM) (6). Moreover, PrEP has emerged as a controversial issue amongst those affected by HIV(8, 9) and there are clear global inequalities in terms of access to PrEP as it is still only available off-label in a number of countries,..

There is a need to understand how uptake and use of PrEP as an HIV prevention strategy could be affected by a range of factors, such as community attitudes towards PrEP and the role of PrEP within concurrent HIV prevention strategies of potential PrEP users (10, 11). Here we report on the first qualitative study in the UK of the acceptability of PrEP amongst populations most affected by HIV to inform targeted PrEP implementation strategies. .

Methods

This article describes a mixed qualitative methods study on the acceptability of PrEP and Treatment as Prevention (TasP) with two communities in Scotland: 1) MSM and 2) men and

For peer review only - http://bmjopen.bmj.com/site/about/guidelines.xhtml

women from migrant African communities (first generation immigrants including asylum seekers, students and other migrants). HIV is largely concentrated in these two communities UK, as they represent the highest levels of HIV prevalence (12, 13). This article reports on findings in relation to barriers to PrEP use. Our sample included HIV-negative and/or untested participants as potential PrEP users and HIV-positive participants as the potential or existing sexual partners of PrEP users. We include findings from HIV-positive participants because of evidence which indicates that serodiscordant sexual partnerships may be an important factor in PrEP acceptability (14). We did not explicitly work with serodiscordant couples so as not to exclude potential acceptability factors and to gather wider community attitudes towards PrEP.

First, exploratory focus groups (FGs) were conducted with a convenience sample of MSM and African participants between August and November 2012 to identify community attitudes and emerging issues around PrEP acceptability. We conducted seven FGs: five with MSM (n=22), and two mixed sex groups with African participants (n=11). Three FGs were conducted with HIV-positive MSM (n=14) and one FG with HIV-positive Africans (n=8). Participant age ranged between 18 and 75 years and discussions took place in urban and semi-urban locations across central Scotland. Participants were recruited through existing community and/or support groups with the assistance of sexual health and/or LGBT organisations. FG discussion topics included existing risk management strategies in sexual health, use of sexual health technologies and exploration of PrEP and TasP (See Table 1).

We then conducted 34 in-depth interviews (IDIs) between March and September 2013 with a purposive sample of MSM (n=20) and African participants (n=14) to explore issues emerging from FG findings and examine personal risk management practices in further depth. Half of

For peer review only - http://bmjopen.bmj.com/site/about/guidelines.xhtml

the IDI participants were HIV-negative or untested for HIV, and the other half had been diagnosed with HIV at the time of the interview (MSM, n=10; Africans, n=7). Inclusion criteria did not specify risk behaviour to allow for findings in relation to candidacy . IDI participants were aged 19 –60 years and were resident in four Scottish regions (Glasgow, Lothian, Lanarkshire, and Grampian). Tailored flyers and posters were distributed to: workshops and support groups; community sexual health testing clinics; gay bars, saunas and clubs; an MSM mail-out condom programme; commercial venues (e.g. African food and barber shops); and in educational settings known to have large African student populations. Community organisations also made contact with potential participants through their regular online and face-to-face outreach and support work. Interviews took place in private spaces in partner organisations or in participants' own homes. The first part of the semi-structured interview focused on personal experiences of HIV, sexual health risk management practices, and use of sexual health technologies. The second part of the interview focused on the acceptability of PrEP and TasP. This included exploring awareness, potential use, concerns and combination with other and/or existing risk management strategies (such as TasP, serosorting, etc) (See Table 2).

PrEP was explained to participants by drawing on but not limited to the use of a visual aid (Figure 1). Basic explanations of PrEP were consistent across all FG and IDI discussions (Table 2). Subsequent and more detailed descriptions of PrEP varied depending on participant questions, which were encouraged and answered. This approach was taken to identify how PrEP should be described to potential candidates. Material did not specify an exact efficacy rate due to the emerging clinical data, variability according to adherence, and to not overly complicate the explanation. Participants were informed that PrEP efficacy was dependent on levels of adherence, as demonstrated in a number of trials. FG participants

For peer review only - http://bmjopen.bmj.com/site/about/guidelines.xhtml

were told that the iPrEx study reported approximately 73% protection if taken regularly (90% adherence), which was accurate at the time discussions were conducted (15). IDI participants were informed that efficacy could be up to or more than 90% if taken regularly, drawing on subsequent sub-analyses of clinical findings (2). Participants were informed that other forms of risk reduction were recommended, such as condoms (16) . Efficacy of condoms was described as less than 100% (18). Discussions explored a wide-range of PrEP scenarios, including non-condom use.

Written consent was provided by all participants at the start of the FGs and IDIs. All FGs and IDIs were digitally recorded and transcribed verbatim. Transcripts were anonymised and coded in NVivo 10. Data were analysed thematically, drawing on both anticipated and emergent themes (19-21). Rigour throughout the analysis was achieved through an iterative process of discussion and revision between co-authors (19, 20, 22). We have identified extracts taken from FGs; otherwise, it can be assumed that the extract comes from an IDI participant. Ethical approval was obtained from the University of Glasgow's College of Social Sciences Ethics Committee (CSS2012/0193, CSS20120264).

Results

We identified five potential barriers to effective PrEP use: interpreting effectiveness; managing adherence; PrEP candidacy and low perceptions of HIV risk; concerns with other risks such as the criminalization of HIV transmission and STIs; and moral barriers.

Interpreting effectiveness

For peer review only - http://bmjopen.bmj.com/site/about/guidelines.xhtml

Understandings of PrEP effectiveness emerged as an important barrier to potential and effective use. Although participants were informed that PrEP was highly effective when taken regularly, most participants expressed concerns that it provided less than 100% protection and therefore was 'insufficient' to prevent HIV transmission on its own. Some participants felt that PrEP used in isolation was 'too much' of a risk (if only approximately 70% effective).

Respondent 1*: That's not enough, that's not enough, exactly.*

Respondent 2: *Its seventy-two per cent effective but then there's still that twenty-eight per cent.*

(HIV-Negative MSM, FG)

Participants also expressed a wider scepticism of PrEP, which emerged in two ways. Some thought that the advice to use condoms with PrEP indicated a continued scientific uncertainty: '*I don't know if this [will] work because since they said if you use it you can still use condom, that means they are not sure as well'* (HIV-negative African woman). Second, some expressed scepticism in relation to the variability in reported efficacy rates in relation to adherence. As a result, there was a reluctance to trust these, unless the information came from a recognised, trusted and reliable source:

R1: *There's so much out there right, on the internet an' everything else right, if it came up on the news right? The logistic news on BBC one that this pill will prevent so…—like a hundred per cent – that's when I would believe it would work. I [would not] trust any other pill …*

Question: *So it has to be a hundred per cent?*

R2: *'cause there's a lot a... no like—anyone can put something on the internet,... You've got to use like certain websites, you've got to look like .org, .gov, those type o'*

For peer review only - http://bmjopen.bmj.com/site/about/guidelines.xhtml

websites. You've just done one that's like www.medicine.co.uk it's not a... it's not like a legitimate website it could just be anyone postin' stuff up there.

***Q:** Well I suppose in the States it's from the Food and Drug Administration so it's got that stamp on it.*

***R1**: Yes, but you [don't know] if they're been a hundred per cent accurate on their job or just been lazy an' the fact that we're brave enough to take it to human trials right?*

(HIV-Negative MSM, FG)

In spite of being told that the FDA had approved the drug, these FG participants did not recognise the organisation or trust it as a reliable source, and were therefore sceptical about its statements and/or endorsement.

Other participants were less concerned about PrEP not being 100% efficacious because they imagined that it would be used *in addition* to condoms. However, participants expressed confusion or uncertainty in knowing how to interpret efficacy rates and their impact on risk reduction in this particular context. For example, in discussing the effectiveness of condoms in relation to other prevention strategies, one participant calculated the protection offered by PrEP, condoms and TasP with a hypothetical HIV-positive sexual partner. Assuming that PrEP was 90% effective at reducing HIV transmission, he explained:

***R**: but there's obviously still a 10% risk but, as you said, there's the same risk with condoms. So it's either you take the 10% risk or you say 'well, I'll use condoms and we'll use TASP' which makes 180%. (Laugh)*

***Q**: And PrEP. (Laugh)*

***R**: 253 out of 300!*

(HIV-negative MSM)

For peer review only - http://bmjopen.bmj.com/site/about/guidelines.xhtml

Although the participant in this extract was joking about adding up the numbers, the interpretation of efficacy rates in relation to other prevention options posed a potential source of confusion or misinformation for participants.

Managing adherence

Given the evidence concerning the patterning of efficacy by adherence (2), maintaining regular adherence to medication was identified as a potential barrier to effective PrEP use. Some participants described how they might forget to take tablets or their routine might be disrupted because of non-regular working patterns, and were therefore concerned about how effective PrEP would be if they did not take the drugs regularly: '*Sometimes I forget. Because I don't have a regular sleeping pattern, so sometimes I'll fall asleep at four in the afternoon and I usually take my pills [for an existing health condition] after dinner'* (HIV-Negative MSM). Establishing and maintaining a routine to take PrEP was also affected by the perception of social stigma attached to HIV medication. This meant maintaining daily adherence would be difficult if there was limited privacy (e.g. the presence of room-mates) or if there was a change in environment. One participant reported concerns about his family potentially finding the tablets:

> *...if I was on those medications for a year and if I went home...or anything like that I'd find it very difficult to be able to take my medications or I would find it a bit of a barrier that if my family knew about it they'd investigate why are you on these pills. And again that would probably put some doubt in their head and then they'd probably then think the worst - that I was HIV positive.*
>
> (HIV-negative MSM)

For peer review only

In addition to being unable to establish or maintain a regular routine to facilitate good adherence, most participants expressed concerns about the physical effects of the drugs themselves, and how the side-effects might inhibit taking them. Some participants described disliking taking tablets and that this would be a barrier to daily PrEP use: *I don't really like pills so I don't think I would take any pills every day'* (HIV-negative African man). Many viewed the potential for side effects, such as nausea and diarrhoea, as too great a trade-off for increased HIV prevention, in spite of being informed that trials had reported high tolerance of the drugs:

> *But, could I put up wi' side-effects all the time as well, like taking a pill, and going tae a nightclub, and ended up wi' diarrhoea? You know, you've gotta think of things like that, am I [going to] take this? Am I [going to] stand tired, have a headache in a club just at the fact that I might at the end of it get a shag...*(HIV-Negative MSM)

Other participants felt the side-effects would interfere with sexual practice itself: '*Nausea, an' diarrhoea an'—you'll be shittin' all over his [penis]. No [thanks].'* (HIV-negative MSM, FG) A significant minority of both HIV-positive and HIV-negative participants raised the issue of longer-term side-effects, especially those who were more familiar with the effects of ARVs:

> *Does it not have any side effects inside my body? 'Cause from my reading as well, some of these anti-retrovirals have got side-effects with the lungs, with the kidneys and stuff. So, in the long run, if I get used to drinking this pill and I'm actually not exposed because I'm thinking I might be at risk of getting exposed, am I not doing more damage to myself in my body?* (HIV-Negative African woman)

Many participants described scepticism in taking PrEP daily, especially if they were not

For peer review only - http://bmjopen.bmj.com/site/about/guidelines.xhtml

always exposed to or at risk of HIV transmission.

PrEP candidacy and low perceptions of HIV risk

Perceptions of HIV transmission risk played an important role in potential uptake of PrEP. Several participants rejected the use of PrEP because they perceived themselves to be at a very low risk. For the majority of HIV-negative participants, this was because most could not imagine knowingly having an HIV-positive sexual partner: '*I wouldn't just [want to] take a risk because it's already defining as a risk, I mean, it's a risk, it's already been defined as a risk and since I'm a little bit risk averse... I won't just do anything'* (HIV-negative African man). Some HIV-negative participants assumed that HIV-disclosure to sexual partners '*was the law'* (HIV-negative MSM, FG), and that their sexual partners were not HIV-positive. Those HIV-negative participants in sero-discordant relationships and HIV-positive participants suggested that condoms, and the nature of their relationships (e.g. monogamous), already helped manage their risks, and that the additional benefit from PrEP was unnecessary. One man in a sero-discordant relationship explained:

> *Right now in my current situation as, I'm doing a monogamous relationship and everything like that, again I don't think even in that [instance] I don't think I would take the pills even though it would be an extra measure...I think I would feel comfortable enough in the current situation.* (HIV-Negative MSM)

PrEP and concerns with other risks

Both HIV-positive and HIV-negative participants identified risk of other sexually-transmitted infections (STIs) as a concern for both themselves and their sexual partners. Heterosexual African participants, especially African women, also talked of the risk of pregnancy. For

For peer review only - http://bmjopen.bmj.com/site/about/guidelines.xhtml

those participants who were either living with HIV, or who had experience of sero-discordant relationships, other health risks posed to the HIV-positive sexual partner through PrEP use were also identified:

> *when I'm looking at condom use it's not just HIV that you're protecting yourself against but some other STIs as well. So you're killing two birds with one stone, more like it. But when it's now using this, you're actually getting yourself involved in something that you already know – 'this person is positive and I'm [going to] have sex with him, but it's okay, I will not use condoms because I know I can use this.' But you're also putting that person, because that person's immune system might be weak. They're also putting that person at risk of other STIs as well, instead of using condoms. So I think it makes people more ignorant of the other things as well. And it makes people just more focused on just HIV and not other STIs. And then risk of pregnancy as well is likely to get high, provided the people are not on any other form of contraception.... No, I think... no, from the way you've said it to me... I wouldn't even tell it to people* (HIV-negative African woman)

Most HIV-positive participants described anxieties about PrEP in relation to *their* inability to be in effective control of HIV prevention. One woman could not imagine agreeing to her HIV-negative sexual partner using PrEP: *'[PrEP] is too risky for him because I don't know when he stop using it, what will happen to him'* (HIV-positive African woman, FG).

Moral barriers to PrEP

PrEP emerged as a highly contentious issue because of the perceived negative implications it had for existing risk management strategies and HIV prevention. Many participants viewed PrEP as problematic because they perceived that *others* would stop using condoms if PrEP

For peer review only - http://bmjopen.bmj.com/site/about/guidelines.xhtml

was available. That is, they distinguished between how *they* might use PrEP and how they perceived others would use it. For some, the concern was in relation to reduced condom use: '*it'll be like... women burning their bras. It'll be all these guys [whipping] off their condoms, do you know what I mean?'* (HIV-negative MSM). Other participants were also concerned with bigger changes in sexual practice, such as increased risk taking:

> *Are you trying to eliminate the condom?... There is no place for a condom at all when it's like this. So I think it actually encourages people to be more promisc... not promiscuous, but to be more... I don't know [if] 'ignorant' is the right word, but to be less careful 'cause they know 'oh, there's that pill'. And it's not even cost-effective than just getting a condom or using...PEP once in a blue moon.* (HIV-negative African woman)

Not all participants were opposed to PrEP and many viewed PrEP as a good addition to HIV prevention options. However, there was still concern about *how* PrEP would be accessed. Overall it was hoped that it would be made available only under strict conditions to ensure the 'right' people received it and that it was used in the 'correct' way:

> *I believe it should be available in Scotland. But, it should be under strict control to make sure the person who takes it knows that he, or she is not protected to full extent from other infections, and HIV either.* (HIV-negative MSM)

Discussion

This is the first qualitative study in the UK to report on the acceptability of PrEP. Our research offers new insights into the psychological and social barriers to PrEP uptake and use. We identified the significance of how the effectiveness of PrEP as a risk reduction

For peer review only - http://bmjopen.bmj.com/site/about/guidelines.xhtml

intervention is communicated to and understood by potential candidates. We demonstrated the importance of HIV risk perception and found that, for many participants, PrEP was not immediately seen as a trusted and/or beneficial addition to their repertoire of existing risk reduction practices. Our findings also highlight how existing risk management strategies in relation to PrEP encompass broad concerns relating to sexual health, relationships, social factors and communities.

Understanding how to interpret PrEP efficacy rates, both on their own and in combination with other prevention strategies, proved a stumbling block for the participants. Liu *et al* identify accurate consumer knowledge as key to PrEP implementation, in addition to addressing other factors such as stigma, adherence, and risk reduction (23). While we agree with Liu, our findings suggest that the form and delivery of this consumer knowledge needs further attention to support effective PrEP use. Negotiating PrEP as a prevention strategy, either on its own or with other strategies, will require improved levels of HIV literacy to be effective. We suggest the need to consider *critical* HIV literacy skills, which encompass the ability to know, understand and use HIV-related information within existing risk reduction practices(24). Our findings suggest that HIV literacy is directly affected by geography, migration status, and sero-status and that inequalities in HIV literacy within communities affected by HIV will play an important role in understanding the barriers to PrEP use. These factors have direct implications for both the nature of how PrEP-related HIV risk prevention is delivered and by whom.

For many participants, PrEP was not seen as a necessary or welcome addition to their repertoire of risk management strategies. Our findings suggest that risk perception and candidacy will play a critical role in decisions to use PrEP, a finding echoed by Golub *et al*

For peer review only - http://bmjopen.bmj.com/site/about/guidelines.xhtml

For peer review only

(25). For some of the HIV-negative participants in our study, the rejection of PrEP as unnecessary emerged from a perception that they were not at risk of HIV transmission. HIV risk was managed often through the sexual exclusion of HIV-positive sexual partners or through reliance on monogamous sexual relationships. These findings suggest that PrEP implementation strategies will need to engage with these wider, socially embedded risk reduction practices. Moreover, our research demonstrates how the management of risk was not limited only to HIV transmission, but also to risk of STIs, pregnancy and social stigma. Participants described how PrEP would not adequately address existing risks, and had the potential to create significant new risks. Saberi *et al* reported similar moral concerns about the implications of PrEP on condom use in their study with MSM participants in serodiscordant relationships in the US (26). Although they surmise that proximity to HIV and age play a role in these concerns, our findings suggest that moral objections to PrEP were not limited by age or sexuality. We suggest that these moral reactions to PrEP as a risk reduction option are related to broader social and community concerns about the potential for PrEP to radically change the way prevention is practiced (9). This highlights the need to engage with wider social concerns about what constitutes ‘inappropriate’ or high-risk sexual practice in relation to PrEP and to demonstrate how PrEP implementation can be a part of a safe and comprehensive risk management strategy.

Our study has a number of strengths and limitations. We employed rigorous qualitative methodology which enabled in-depth exploration of the social meanings of PrEP acceptability and likely use (21). We therefore add to existing quantitative PrEP acceptability research. With a small sample of MSM and migrant African participants in a non-generalized HIV epidemic., some of whom were engaged in sexual health or community services, our findings are not generalisable to a wider population but we would argue, are

For peer review only - http://bmjopen.bmj.com/site/about/guidelines.xhtml

transferable to similar populations in similar social contexts. As we did not sample according to sexual risk behaviour, our findings are only transferrable to broad risk groups and not necessarily to 'high risk' individuals. Our inclusion of the recommendation to use condoms in the visual PrEP information may have biased the findings. However, our discussions included consistent and broad descriptions of PrEP and encompassed a wide range of PrEP scenarios, including non-condom use.

Our research demonstrates the need to consider acceptability factors which extend beyond drug adherence and risk compensation when introducing and scaling up PrEP and has a number of implications for policy and clinical practice. In particular, it will be necessary to develop clear tools and techniques to communicate PrEP information to potential candidates. These methods will need to translate clinical research relating to PrEP effectiveness in real world contexts as is appropriate in the context of diverse critical literacy skills. Implementation will also need to address low risk perception, non-HIV risk reduction and other moral concerns to demonstrate how PrEP can be a part of a safe and comprehensive risk management strategy. Our study suggests that broader social and community concerns about the potential for PrEP to change HIV prevention need to be addressed by supporting the integration of PrEP into existing risk management strategies and through targeted promotion.

For peer review only

For peer review only - http://bmjopen.bmj.com/site/about/guidelines.xhtml

Author's Contributions

IY designed the study, carried out the qualitative data collection and analysis and drafted the manuscript. PF helped to design the study, carried out some of the data collection and analysis and helped to draft the manuscript. LMD participated in the design of the study and helped to draft the manuscript. All authors read and approved the final manuscript.

Acknowledgements

The HIV and the Biomedical Study, IY and LMD are core funded by the UK Medical Research Council (MRC) (MC_U130031238/MC_UU_12017/2) at the MRC/Chief Scientist Office (CSO) Social and Public Health Sciences Unit, University of Glasgow. PF is funded by Glasgow Caledonian University. We thank the organisations (Waverly Care, Terrence Higgins Trust Scotland, LGBT Youth Scotland, Gay Men's Health Scotland, Positive Scotland, NHS Greater Glasgow and Clyde, NHS Lothian, NHS Grampian) who helped with recruitment and the men and women who agreed to take part in the focus groups and interviews.

Data Sharing Statement

This study was undertaken by the MRC/CSO Social and Public Health Sciences Unit, University of Glasgow. Our data sharing policies comply with that of the UK Medical Research Council. Unpublished data from this study is available upon request and information about the study and data is made available on the MRC/CSO Social and Public Health Sciences website. https://www.sphsu.mrc.ac.uk/research-programmes/sh/shfsub/accbiomhivprev.html

Competing Interests

We have no competing interests.

For peer review only

References

1. Baeten JM, Grant R. Use of Antiretrovirals for HIV Prevention: What Do We Know and What Don't We Know? Current HIV/AIDS Reports. 2013;10(2):142-51.

2. Anderson PL, Glidden DV, Liu A, et al. Emtricitabine-Tenofovir Concentrations and Pre-Exposure Prophylaxis Efficacy in Men Who Have Sex with Men. Science Translational Medicine. 2012;4(151):1-8.

3. Young I, McDaid L. How Acceptable are Antiretrovirals for the Prevention of Sexually Transmitted HIV?: A Review of Research on the Acceptability of Oral Pre-exposure Prophylaxis and Treatment as Prevention. AIDS Behav. 2014 Jul 30;18 (2):195-216.

4. Holt M. HIV pre-exposure prophylaxis and treatment as prevention: a review of awareness and acceptability among men who have sex with men in the Asia-Pacific region and the Americas. Sex Health. 2013 Jul 19.

5. Gamarel KE, Golub SA. Intimacy Motivations and Pre-exposure Prophylaxis (PrEP) Adoption Intentions Among HIV-Negative Men Who Have Sex with Men (MSM) in Romantic Relationships. Annals of Behavioral Medicine. 2014:1-10.

6. Kirby T, Thornber-Dunwell M. Uptake of PrEP for HIV slow among MSM. Lancet. 2014;383(9915):399-400.

7. Amico KR, Stirratt MJ. Adherence to Preexposure Prophylaxis: Current, Emerging, and Anticipated Bases of Evidence. Clinical Infectious Diseases. 2014 July 1, 2014;59(suppl 1):S55-S60.

8. Cairns G. The road to PrEP: trials, regulation and roll-out. aidsmap. 2012.

9. Belluz J. The Truvada wars. BMJ. 2014(348):g3811

For peer review only

10. Kippax S, Stephenson N. Beyond the distinction between biomedical and social dimensions of HIV prevention through the lens of a social public health. Am J Public Health. 2012 May;102(5):789-99.

11. Nguyen VK, Bajos N, Dubois-Arber F, et al. Remedicalizing an epidemic: from HIV treatment as prevention to HIV treatment is prevention. AIDS. 2011 Jan 28;25(3):291-3.

12. Health Protection Scotland. ANSWER: HIV infections and AIDS in Scotland: Quarterly report to 30 June 2013: Health Protection Scotland2013 4 September 2013 Contract No.: 2013/36.

13. PHE. HIV in the United Kingdom: 2013 Report. London: Public Health England2013.

14. Ware NC, Wyatt MA, Haberer JE, Baeten JM, et al. What's love got to do with it? Explaining adherence to oral antiretroviral pre-exposure prophylaxis for HIV-serodiscordant couples. J Acquir Immune Defic Syndr. 2012 Apr 15;59(5):463-8.

15. Grant RM, Lama JR, Anderson PL, et al. Preexposure chemoprophylaxis for HIV prevention in men who have sex with men. New England Journal of Medicine. 2010;363(27):2587-99.

16. Center for Disease Control (CDC). Interim Guidance: Pre-exposure Prophylaxis for the Prevention of HIV Infection in Men Who Have Sex with Men. Morbidity and Mortality Weekly Report. 2011;60(3):65-8.

17. Cairns G. Consistent condom use in anal sex stops 70% of HIV infections, study finds. 4 March 2013 [10 September 2014]; Available from: http://www.aidsmap.com/Consistent-condom-use-in-anal-sex-stops-70-of-HIV-infections-study-finds/page/2586976/.

18. Weller S, Davis K. Condom effectiveness in reducing heterosexual HIV transmission. Cochrane Database Syst Rev. 2002;1(1).

19. Mason J. Qualitative Researching. London: Sage Publications; 1996.

For peer review only - http://bmjopen.bmj.com/site/about/guidelines.xhtml

20. Barbour R, Kitzinger J, editors. Developing Focus Group Research: Politics, Theory and Practice. London: Sage Publications; 1999.

21. Ziebland S, McPherson A. Making sense of qualitative data analysis: an introduction with illustrations from DIPEx (personal experiences of health and illness). Medical Education. 2006;40(5):405-14.

22. Silverman D. Doing Qualitative Research: A Practical Handbook. London: Sage; 2000.

23. Liu A, Cohen S, Follansbee S, Cohan D, et al. Early Experiences Implementing Pre-exposure Prophylaxis (PrEP) for HIV Prevention in San Francisco. PLoS Med. 2014;11(3):e1001613.

24. Sørensen K, Van den Broucke S, Fullam J, et al. Health literacy and public health: a systematic review and integration of definitions and models. BMC Public Health. 2012;12(1):80.

25. Golub SA, Gamarel KE, Rendina HJ, et al. From efficacy to effectiveness: facilitators and barriers to PrEP acceptability and motivations for adherence among MSM and transgender women in New York City. AIDS patient care and STDs. 2013;27(4):248-54.

26. Saberi P, Gamarel KE, Neilands TB, et al. Ambiguity, ambivalence, and apprehensions of taking HIV-1 pre-exposure prophylaxis among male couples in San Francisco: a mixed methods study. PLoS One. 2012;7(11):e50061.

For peer review only

For peer review only - http://bmjopen.bmj.com/site/about/guidelines.xhtml

Table 1: Focus Group Discussion Guide

Part 1

Participants were presented with a number of objects to discuss. Objects included: condoms, sachets of lubricant, pregnancy test, aidsmap list of ARVs, mocked up bottle of antibiotics, empty boxes/bottles of Truvada, and pictures of: Orasure quicktest and rapid HIV test

- **What do these objects make you think about?**
  - i.e. engagement with sexual health organisations? Own HIV status? Sexual encounters? Pleasure? Community? Privacy?

- **How do these objects relate to risk and HIV?**
  - Who uses these objects? Do you use these objects? Why, or why not?

- **What is risky in relation to HIV?**
  - Is it the person, the sexual position, the location, your own health?

- **Do you use these objects to manage HIV?**
  - If yes, how? If not, why not?
  - Does using these objects hinder/help management of HIV/disclosure of status to others?

- **What else do you use to manage HIV?**
  - i.e. how do you assess risk and how do you manage it? Gaydar profile? Looks?

Part 2

Provide cards of PrEP & TasP provided & explain separately.

- **How might you use these pills?**
  - Ask about serodiscordant partnerships and how these pills might change how HIV transmission is managed?
  - Would the potential use of these pills change depending on: sexual partner, current health experiences, location (space, place), etc?

- **How do you think your friends or sexual partners might use these pills?**

For peer review only

- Would people choose these pills? Would they choose them in combination with condoms? Would they trust these pills would work? Would this depend on the sexual partner?
- How would you decide to use them? How do you decide on current sexual health practices?

- **Are you concerned about the use of pills as a form of HIV prevention?**
  - Concerns about other STIs/Hep C?
  - Would the side effects be a problem? What are your current experiences with side effects and do you think other men could manage these?

- **Would these pills change the way people currently manage HIV? What do you think about this?**
  - Ask about understandings/experiences of condom use, feelings of security/management of risk with existing options?
  - Would this increase/decrease HIV related stigma?
  - Would this change where the onus of responsibility was placed within the community? (i.e. are HIV positive people more or less responsible to manage transmission now, and would these pills/interventions change things?)

For peer review only

For peer review only - http://bmjopen.bmj.com/site/about/guidelines.xhtml

Table 2: Interview Topic Guide for HIV-negative or Untested Participants

1) Experiences with/proximity to HIV

Before I start asking you questions specifically about the latest developments, I'd like to learn a little more about your own experiences with HIV. Can you tell me about that?

Confirm HIV Status: HIV negative/unknown status/undisclosed/other

- Is HIV a risk for you?
  - If yes, what are you concerned about in particular?
  - If you are not concerned about HIV, why not?
  - Has this changed at all for you? (different sexual partners, location, sexual practice, etc)
- Is HIV something that you talk about with your sexual partners? (or friends?)
  - If yes, what do you speak about? In what contexts would you talk about HIV?
  - If not, would you feel able to discuss HIV or any concerns you had about HIV with your sexual partners and/or friends?
- Have you ever tested for HIV?
  - If yes:
    - What prompted you get a test?
    - Where did you get the test done?
    - Have you tested for HIV more than once? How often do you get tested?
  - If not:
    - Have you thought about getting tested before?
    - Is there anything that's stopped you from seeking an HIV test?
    - What might make you get tested?

2) Risk management/sexual health

This study is about understanding how people manage their sexual health and risk of HIV.

- What are the risks for you in relation to sexual health?
- You've said that you manage risk in sexual health by ….;
  - Has this changed over time?
  - Does this change according to sexual partner?
  - Does this change according to location? (home, travelling, public spaces)

For peer review only - http://bmjopen.bmj.com/site/about/guidelines.xhtml

- Do you talk to your sexual partners about this?
    - Why/Why not?
- Do you use health or other services to help you manage your sexual health? If so, how?

3) **Use of existing technologies** –

I'm interested in how people use existing sexual health technologies. This can include lots of things. Here is a list of some of the things that people might use.

List of sexual health technologies presented to participant:

condoms
HIV testing
Sexually transmitted infections (STI) testing
Contraception (the pill, IUD, long-term injections, etc)
pregnancy testing
CD4 counts
viral loads
Anti-retrovirals (ARVs)
post-exposure prophylaxis (PEP)

- Do you use any of these now? Have you used any of these in the past?
    - If not, is there anything that is missing from this list that you or people you know have used?
- How have you used them? What made you use them?
    - How do they work? (explore how is this related to understandings of risk of HIV transmission)
- Have you used any of these in combination with other prevention methods?
- How do you feel about using them?
    - Ease/difficulty of using these?
    - Social concerns?
    - Trust in effectiveness?
- If you started/stopped using some of these, can you say why you did?

4) **Potential use of new technologies**

For peer review only - http://bmjopen.bmj.com/site/about/guidelines.xhtml

This study is about exploring how people feel about using ARVs, the medication currently used to treat HIV, as a way of preventing HIV. This can be done in a couple of ways.

PrEP

Provide card, but verbally explain:

- *PrEP*– Pre-exposure Prophylaxis (PrEP) is when ARVs are used by people who are HIV negative to prevent the transmission of HIV.
- At the moment, it can be taken once a day, although researchers are looking into other forms (short term PrEP, long-acting injectable, topical gel/microbicides).
- PrEP only works if people take the medication regularly. Clinical trials have shown that it is effective in relation to how often people take the pills.
- People are still encouraged to use condoms and other forms of risk reduction with PrEP use.
- There are some side effects, but this may not affect everyone and it generally seems to be well tolerated in the clinical trials.
- PrEP is not currently available in the UK, but it has been licensed for use in the United States.

- Have you heard of PrEP before?
    - If so, where? From whom? What had you heard?
- What do you think of PrEP as a prevention method?
- How would you feel about using PrEP as a prevention method?
    - If would not, why not?
    - If good, why?
- If you would use PrEP, how do you think you would you use it?
    - In combination with other forms of prevention? As a back-up?
    - In what circumstances might you see PrEP as a potential prevention method?
- Do you have any concerns about PrEP as a prevention method?
    - Concerns about taking medication to prevent HIV transmission? (interactions, remembering tablets, side effects, etc)
    - Do you have any concerns about how it might work?
    - Would there be any social stigma associated with PrEP?
- Do you think other people might use PrEP as a method?
    - What do you think about that?

TasP

For peer review only - http://bmjopen.bmj.com/site/about/guidelines.xhtml

Provide card, but verbally explain:

- Treatment as prevention is when ARVs are used by people living with HIV to not only to clinically manage HIV, but also to help prevent the transmission of HIV.
- TasP manages the 'viral load' or the amount of HIV in the system. Research has show that having an 'undetectable' viral load means that transmission of HIV is unlikely to happen. So, if the HIV positive person is taking their treatment regularly, and they don't have an STI, and their viral load is 'undetectable' for a period of time (about 6 months), they would clinically be considered not infectious.
- TasP in particular is when treatment is started to prevent transmission, rather than when the person clinically needs the treatment.
- If someone starts this treatment early – including for prevention reasons – they cannot stop taking this medication. So although people living with HIV will eventually move onto treatment, perhaps after 5 to even 10 years, this would mean starting treatment considerably earlier.

- Have you heard of TasP before?
    - If so, where? From whom? What had you heard?
- What do you think of TasP as a prevention method?
    - What do you think about using drugs to lower levels of infection in people living with HIV?
- Can you imagine using this as a prevention method with a sexual partner who is HIV positive?
    - If yes, why?
    - If not, why not?
- How would you feel if a sexual partner suggested this as an HIV prevention method?
- Do you have any concerns about this as a prevention method?
    - Trust in viral load levels/science?
    - Concerns with social perceptions? (i.e. social stigma around ARVs)
    - Coercion/pressure by sexual partners not to use condoms?
    - Anxieties around STIs?
- How do you think other people who are HIV negative or untested might feel about using ARVs or HIV treatment as a prevention method?
    - What do you think about that?

General themes

In addition to the above themes/areas of enquiry, the interview will pay particular attention to cross-cutting themes and issues such as:

- Gender and sexuality

For peer review only - http://bmjopen.bmj.com/site/about/guidelines.xhtml

- Social stigma (HIV-related or otherwise)
- HIV & sexual health literacy
- HIV serostatus as an identity (or not) and how this might function/influence social practices
- Community norms and practices in relation to sexual relations and sexual health
- Social and structural barriers/enablers to sexual health & use of technologies (i.e. access to services, cost, stigma, understandings of rights, legal issues, etc)
- Individual vs group rights and responsibilities
- Trust in science, trust in people

For peer review only

For peer review only - http://bmjopen.bmj.com/site/about/guidelines.xhtml

Barriers to uptake and use of Pre-Exposure Prophylaxis (PrEP) amongst communities most affected by HIV in the UK: Findings from a qualitative study in Scotland

Ingrid Young, Paul Flowers, Lisa McDaid

Running Head: Barriers to Pre-Exposure Prophylaxis (PrEP) in ~~Scotland~~the UK

Authors: IYoung[1], P Flowers[2], LM McDaid[1]

Key Words: Pre-Exposure Prophylaxis (PrEP), men who have sex with men (MSM), migrant African communities, Scotland, biomedical HIV prevention

Affiliation:

1. MRC/CSO Social and Public Health Sciences Unit, University of Glasgow
2. School of Health and Life Sciences, Glasgow Caledonian University

Corresponding Author:

Ingrid Young,
MRC/CSO Social and Public Health Sciences Unit, University of Glasgow
200 Renfield Street
Glasgow, G2 3QB
0141 353 7533
ingrid.young@glasgow.ac.uk

For peer review only

Abstract

Objectives: To explore the acceptability of Pre-Exposure Prophylaxis (PrEP) amongst gay, bisexual and men who have sex with men (MSM) and migrant African communities in Scotland, UK.

Design: Consecutive mixed qualitative methods ~~using~~ consisting of focus groups (FGs) and in-depth interviews (IDI) ~~were conducted to explore the acceptability of PrEP~~ explored PrEP acceptability. Data were digitally recorded, transcribed and analysed thematically to identify anticipated and emerging themes.

Setting: Participants were recruited through community sexual health and~~&~~ outreach support services, and from non-sexual health settings across ~~urban and semi-urban~~ Scotland.

Participants: Inclusion criteria included: identification as either MSM and/or from migrant African communities; 18 years and older; living in Scotland at the time of participation. 7 ~~focus groups~~FGs were conducted (n=33): 5 with MSM (n=22), and 2 mixed-sex groups with African participants (n=11, women=8), aged 18 – 75 years. 34 ~~in-depth interviews~~IDIs were conducted with MSM (n=20) and African participants (n=14, women = 10), aged 19 – 60 years. The sample included participants who were HIV-positive and HIV-negative or untested (HIV-positive FG participants ~~in focus groups,~~, n=22; HIV-positive IDI participants ~~in interviews~~, n=17).

Results: ~~Perceptions~~ Understandings of PrEP effectiveness~~,~~ ~~HIV literacy~~ and concerns about maintaining regular adherence were identified as barriers to PrEP uptake and use. Low perception of HIV risk ~~as a result of~~due to existing risk management strategies meant ~~that~~ few participants saw themselves as PrEP candidates. Participants identified risk of other STIs and pregnancy as a concern which PrEP did not address for either themselves or their sexual partners. PrEP emerged as a contentious issue because of the potentially negative implications it had for HIV prevention. Many participants viewed PrEP as problematic

For peer review only

For peer review only - http://bmjopen.bmj.com/site/about/guidelines.xhtml

because they perceived that *others* would stop using condoms if PrEP was to become available.

Conclusions: PrEP implementation will needs to: address identify appropriate communication methods in the context of diverse HIV literacy; address HIV literacy, risk reduction concerns, and; demonstrate how PrEPhow it can be part of a safe and comprehensive risk management strategy.

Article summary/Strengths and limitations of this study

- Our study offers new insights into the psychological and social barriers to PrEP uptake and use.
- This paper identifies how inequalities in critical HIV literacy and wider community attitudes affect PrEP acceptability. highlights the importance of HIV literacy in effective and efficient PrEP uptake and use.
- We demonstrate the need to consider socially embedded sexual risk management strategies, which address concerns beyond risk of HIV transmission.
- We suggest there is a need to consider how clinical research is translated into real world contexts and communicated to potential users, as well as how potential users are supported in integrating this information into existing risk management strategies.

For peer review only - http://bmjopen.bmj.com/site/about/guidelines.xhtml

Pre-Exposure Prophylaxis (PrEP) represents a significant biomedical addition to international HIV prevention efforts. The clinical efficacy of the use of anti-retrovirals (ARVs) by HIV-negative individuals to prevent the sexual transmission of HIV has been widely tested and its clinical efficacy has been extensively reported (1, 2). The acceptability of PrEP to potential PrEP users (or candidates) to date has focused largely, although not exclusively, on the risk of inefficient PrEP use, such as low or inconsistent adherence to the medication and reduced condom use. It has also focused predominately on experiences in the United States (US) (3-5). There are currently a number of PrEP demonstration studies underway in the United Kingdom (UK), Australia, United States (US) and elsewhere to explore how it might be used in 'real world' settings (6, 7)(3). However, the acceptability of PrEP to potential PrEP users (or candidates), has received significantly less attention (4, 5). Most research has focused on the risk of inefficient PrEP use by potential candidates, such as low or inconsistent adherence to the medication and reduced condom use. It has also focused predominately on US experiences. Despite being available in the US since the Food and Drug Administration (FDA) approved Truvada (emtricitabine/tenofovir disoproxil fumarate) for use as PrEP in 2012, there has been relatively low uptake to date amongst targeted communities affected by HIV, including gay, bisexual and other men who have sex with men (MSM) (6)(3). Moreover, PrEP has emerged as a controversial issue amongst those affected by HIV(8, 9)(6) and there are clear global inequalities in terms of access to PrEP as it is still only available off-label is not available in a number of countries, including the U.K.

There is a need to understand the acceptability of PrEP, how the uptake and use of PrEP as an HIV prevention strategy could be affected by a range of factors,, such as the social environment in which it is introduced, community attitudes towards PrEP and and the role of PrEP within concurrent HIV prevention strategies of potential PrEP users (10, 11)(7, 8).

For peer review only - http://bmjopen.bmj.com/site/about/guidelines.xhtml

Here we describe report on the first a qualitative study in the UK of the social barriersacceptability of to PrEP uptake and use amongst populations most affected by HIV in Scotland, UK to inform thetargeted PrEP implementation of PrEPstrategies. in low prevalence settings.

Methods

This article reports ondescribes aWe undertook a mixed qualitative methods study onf the acceptability of PrEP and Treatment as Prevention (TasP) with two communities in Scotland: 1) MSM and 2) men and women from migrant African communities. (Th group was defined as first generation immigrants from sub-Saharan Africa, and includedincluding asylum seekersrefugees, economic migrants and students and other migrants). HIV is largely concentrated in Tthese two communities represent over half of all new HIV diagnoses in Scotland, as well as across the UK, as they represent the highest levels of HIV prevalence (12, 13)(9, 10). This article reports on findings iin relation to barriers to PrEP use. We Our sample included HIV-negative and/or untested participants as potential PrEP users, and and HIV-positive participants as the potential or existing sexual partners of PrEP users. , although we did not explicitly work with sero-discordant couples. We include findings from HIV-positive participantsThe latter group were included because of evidence which indicates that serodiscordant sexual partnerships may be an important factor in PrEP acceptability (14)(11). We did not explicitly work with serodiscordant couples so as not to exclude potential acceptability factors and , and to gather wider community attitudes towards PrEP.

First, exploratory focus groups (FGs) were conducted with a convenience sample of MSM and African men and womenparticipants between August and November 2012 to identify community attitudes and emerging issues in relation to potentialaround PrEP acceptability.

For peer review only - http://bmjopen.bmj.com/site/about/guidelines.xhtml

We conducted seven FGs: five with MSM (n=22), and two mixed sex groups with -African participants (n=11). Three FGs were conducted with HIV-positive MSM (n=14) and one FG with HIV-positive Africans (n=8). The age range of pParticipant ages was ranged between 18 and 75 years and discussions took place across in urban and semi-urban locations across central in Scotland (Glasgow, Edinburgh, Motherwell and Selkirk). Participants were recruited through existing community and/or support groups with the assistance of sexual health and/or LGBT organisations. FG discussion topics included existing risk management strategies in sexual health, use of sexual health technologies (e.g. condoms, testing, ARVs, etc) and exploration of PrEP and TasP biomedical HIV prevention interventions (PrEP and Treatment as PreventionTasP).(See Table 1).

PrEP was explained with the use of visual aids (Figure 1). Material did not specify an efficacy rate, but, when asked, participants were informed that it was dependent on levels of adherence and that the iPrEx study reported approximately 73% protection, which was accurate at the time discussions were conducted (12).

We then conducted 34 in-depth interviews (IDIs) between March and September 2013 with a purposive sample of MSM (n=20) and African men and womenparticipants (n=14) to explore issues emerging from FG findings and examine and personal risk management practices in further depth. Half of the IDI participants were HIV-negative or untested for HIV, and the other half had been diagnosed with HIV at the time of the interview (MSM, n=10; Africans, n=7). Inclusion criteria did not specify risk behaviour so as not to excludeto allow for potential findings in relation to candidacy . IDI participants were aged 19 –60 years and were resident in four Scottish regions (Glasgow, Lothian, Lanarkshire, and Grampian). Tailored flyers and posters were distributed to: workshops and support groups, including meetings and events for people living with HIV; community HIV and STIsexual health testing clinics; gay

For peer review only - http://bmjopen.bmj.com/site/about/guidelines.xhtml

bars, saunas and clubs; an MSM mail-out condom programme for MSM; commercial venues such as (e.g. African food and barber shops); and in educational settings known to have large African student populations. Community organisations also made contact with potential participants through their regular online and face-to-face outreach and support work. Interviews took place in private spaces in partner organisations which werelocations convenient to participants, including private offices, university classrooms, meeting rooms or in participants' own homes. The first part of the semi-structured interview focused on personal experiences of and perspectives on HIV, sexual health risk management practices, and use of sexual health technologies (as before). The second part of the interview focused on the acceptability of PrEP and TasP. This included exploring, focusing on awareness, potential use, concerns and combination with other and/or existing risk management strategies (such as TasP, serosorting, etc). (See Table 2).

PrEP was explained to participants by drawing on but not limited to the use of a visual aid (Figure 1). Basic explanations of PrEP were consistent across all FG and IDI discussions (Table 2). Subsequent and more detailed descriptions of PrEP varied depending on participant questions, which were encouraged and answered. This approach was taken to identify how PrEP should be described to potential candidates. Material did not specify an exact efficacy rate due to the emerging clinical data, variability according to adherence, and to not overly complicate the explanation. Participants were informed that PrEP efficacy was dependent on levels of adherence, as demonstrated in a number of trials. FG participants were told that the iPrEx study reported approximately 73% protection if taken regularly (90% adherence), which was accurate at the time discussions were conducted (15). IDI participants were informed that efficacy could be up to or more than 90% if taken regularly, drawing on subsequent sub-analyses of clinical findings (2). Participants were informed that other forms

For peer review only

For peer review only - http://bmjopen.bmj.com/site/about/guidelines.xhtml

of risk reduction were recommended, such as condoms (16) . Efficacy of condoms was described as less than 100% (18). Discussions explored a wide-range of PrEP scenarios, including non-condom use.

Written consent was provided by all participants at the start of the FGs and IDIs. All FGs and IDIs were digitally recorded and transcribed verbatim. Transcripts were anonymised and coded in NVivo 10. Data were analysed thematically, drawing on both anticipated and emergent themes (19-21)(13-15). Rigour throughout the analysis was achieved through an iterative process of discussion and revision between co-authors (19, 20, 22)(13, 14, 16). We have identified extracts taken from FGs; otherwise, it can be assumed that the extract comes from an IDI participant. Ethical approval was obtained from the University of Glasgow's College of Social Sciences Ethics Committee (CSS2012/0193, CSS20120264).

Results

We identified five potential barriers to effective PrEP uptake and use: interpreting effectiveness and HIV literacy; managing adherence to PrEP; PrEP candidacy and low perceptions of HIV risk; concerns with other risks such as the criminalization of HIV transmission and STIs; and moral barriers.

Interpreting effectiveness and HIV literacy

Perceptions Understandings of PrEP effectiveness emerged as an important significant barrier to potential and effective use. Although participants were informed that PrEP was highly

For peer review only - http://bmjopen.bmj.com/site/about/guidelines.xhtml

effective when taken regularly, ~~M~~most participants expressed concerns ~~about its effectiveness to prevent HIV transmission. These concerns were often grounded in the~~that it provided ~~ ~~less than 100% protection~~efficacy~~ ~~rates ~~and ~~a perception~~therefore ~~ that PrEP provided~~was 'insufficient' ~~protection from~~to prevent HIV transmission on its own. Some participants felt that PrEP used in isolation was 'too much' of a risk (if only approximately 70% effective~~ in clinical trials~~).

> ***Respondent 1**: That's not enough, that's not enough, exactly.*
>
> ***Respondent 2:** Its seventy-two per cent effective but then there's still that twenty-eight per cent.*
>
> (HIV-Negative MSM, FG)

Participants also expressed a wider scepticism ~~with regards to~~of PrEP, which emerged in two ways. Some thought that the advice to use condoms with PrEP indicated a continued scientific uncertainty: '*I don't know if this [will] work because since they said if you use it you can still use condom, that means they are not sure as well'* (HIV-negative African woman). Second, some expressed scepticism in relation to the variability in reported efficacy rates ~~as described ~~in relation to adherence. As a result, there was a reluctance to trust these, unless the information came from a recognised, trusted and reliable source:

> ***R1:** There's so much out there right, on the internet an' everything else right, if it came up on the news right? The logistic news on BBC one that this pill will prevent so…—like a hundred per cent – that's when I would believe it would work. I [would not] trust any other pill ...*
>
> ***Question:** So it has to be a hundred per cent?*
>
> ***R2:** 'cause there's a lot a... no like—anyone can put something on the internet,... You've got to use like certain websites, you've got to look like .org, .gov, those type o'*

For peer review only

For peer review only - http://bmjopen.bmj.com/site/about/guidelines.xhtml

websites. You've just done one that's like www.medicine.co.uk it's not a... it's not like a legitimate website it could just be anyone postin' stuff up there.

Q: *Well I suppose in the States it's from the Food and Drug Administration so it's got that stamp on it.*

R1*: Yes, but you [don't know] if they're been a hundred per cent accurate on their job or just been lazy an' the fact that we're brave enough to take it to human trials right?*

(HIV-Negative MSM, FG)

In spite of being told that the FDA had approved the drug, these FG participants did not ~~know~~ recognise the organisation or trust it as a reliable source, and were therefore sceptical about its statements and/or endorsement.

Other participants were less concerned about PrEP not being 100% efficacious because they imagined that it would be used *in addition* to condoms. However, participants expressed confusion or uncertainty in knowing how to interpret efficacy rates and their impact on risk reduction in this particular context. For example, in discussing the effectiveness of condoms in relation to other prevention strategies, one participant ~~added up~~calculated the protection offered by PrEP, condoms and TasP with a hypothetical HIV-positive sexual partner. Assuming that PrEP was 90% effective at reducing HIV transmission, he explained:

R*: but there's obviously still a 10% risk but, as you said, there's the same risk with condoms. So it's either you take the 10% risk or you say 'well, I'll use condoms and we'll use TASP' which makes 180%. (Laugh)*

Q*: And PrEP. (Laugh)*

R*: 253 out of 300!*

(HIV-negative MSM)

For peer review only - http://bmjopen.bmj.com/site/about/guidelines.xhtml

Although the participant in this extract was joking about adding up the numbers, the interpretation of efficacy rates in relation to other prevention options posed a potential source of confusion or misinformation for participants.

Managing adherence ~~to PrEP~~

Given the evidence concerning the patterning of efficacy by adherence (2), maintaining regular adherence to medication was identified as a potential barrier to effective PrEP use. Some participants described how they might forget to take tablets or their routine might be disrupted because of non-regular working patterns, and were therefore concerned about how effective PrEP would be if they did not take the drugs regularly: '*Sometimes I forget. Because I don't have a regular sleeping pattern, so sometimes I'll fall asleep at four in the afternoon and I usually take my pills [for an existing health condition] after dinner'* (HIV-Negative MSM). Establishing and maintaining a routine to take PrEP was also affected by the perception of social stigma attached to HIV medication. This meant maintaining daily adherence would be difficult if there was limited privacy (e.g. the presence of room-mates) or if there was a change in environment. One participant reported concerns about his family potentially finding the tablets:

> *...if I was on those medications for a year and if I went home...or anything like that I'd find it very difficult to be able to take my medications or I would find it a bit of a barrier that if my family knew about it they'd investigate why are you on these pills. And again that would probably put some doubt in their head and then they'd probably then think the worst - that I was HIV positive.*
>
> (HIV-negative MSM)

For peer review only - http://bmjopen.bmj.com/site/about/guidelines.xhtml

In addition to being unable to establish or maintain a regular routine to facilitate good adherence, most participants expressed concerns about the physical effects of the drugs themselves, and how the side-effects might inhibit taking them. Some participants described disliking taking tablets and ~~reported~~ that this would be a barrier to daily PrEP use: *I don't really like pills so I don't think I would take any pills every day'* (HIV-negative African man). Many viewed the potential for side effects, such as nausea and diarrhoea, as too great a trade-off for increased HIV prevention, in spite of being informed that trials had reported high tolerance of the drugs:

> *But, could I put up wi' side-effects all the time as well, like taking a pill, and going tae a nightclub, and ended up wi' diarrhoea? You know, you've gotta think of things like that, am I ~~gonna~~ [going to] take this? Am I ~~gonnae~~ [going to] stand tired, have a headache in a club just at the fact that I might at the end of it get a shag...*(HIV-Negative MSM)

Other participants felt the side-effects would interfere with sexual practice itself: '*Nausea, an' diarrhoea an'—you'll be shittin' all over his [penis]~~knob~~. No [thanks]~~ta~~.'* (HIV-negative MSM, FG) A significant minority of both HIV-positive and HIV-negative participants raised the issue of longer-term side-effects, especially those who were more familiar with the effects of ARVs:

> *Does it not have any side effects inside my body? 'Cause from my reading as well, some of these anti-retrovirals have got side-effects with the lungs, with the kidneys and stuff. So, in the long run, if I get used to drinking this pill and I'm actually not exposed because I'm thinking I might be at risk of getting exposed, am I not doing more damage to myself in my body?* (HIV-Negative African woman)

For peer review only - http://bmjopen.bmj.com/site/about/guidelines.xhtml

Many participants described scepticism in taking PrEP daily, especially if they were not always exposed to or at risk of HIV transmission.

PrEP candidacy and low perceptions of HIV risk

Perceptions of HIV transmission risk played an important role in potential uptake of PrEP. Several participants rejected the use of PrEP because they perceived themselves to be at a very low risk. For the majority of HIV-negative participants, this was because most could not imagine knowingly having an HIV-positive sexual partner: '*I wouldn't just ~~wanna~~ [want to] take a risk because it's already defining as a risk, I mean, it's a risk, it's already been defined as a risk and since I'm a little bit risk averse... I won't just do anything'* (HIV-negative African man). Some HIV-negative participants assumed that HIV-disclosure to sexual partners '*was the law*' (HIV-negative MSM, FG), and that their sexual partners were not HIV-positive. Those HIV-negative participants in sero-discordant relationships and HIV-positive participants suggested that condoms, and the nature of their relationships (e.g. monogamous), already helped manage their risks, and t~~therefore, they did not think t~~hat the additional benefit from PrEP was unnecessary. One man in a sero-discordant relationship explained:

> *Right now in my current situation as, I'm doing a monogamous relationship and everything like that, again I don't think even in that [instance] I don't think I would take the pills even though it would be an extra measure...I think I would feel comfortable enough in the current situation.* (HIV-Negative MSM)

PrEP and concerns with other risks

For peer review only - http://bmjopen.bmj.com/site/about/guidelines.xhtml

Both HIV-positive and HIV-negative participants identified risk of other sexually-transmitted infections (STIs) as a concern for both themselves and their sexual partners. Heterosexual African participants, especially African women, also talked of the risk of pregnancy. For those participants who were either living with HIV, or who had experience of sero-discordant relationships, other health risks posed to the HIV-positive sexual partner through PrEP use wereas also identified:

> *when I'm looking at condom use it's not just HIV that you're protecting yourself against but some other STIs as well. So you're killing two birds with one stone, more like it. But when it's now using this, you're actually getting yourself involved in something that you already know – 'this person is positive and I'm gonna [going to] have sex with him, but it's okay, I will not use condoms because I know I can use this.' But you're also putting that person, because that person's immune system might be weak. They're also putting that person at risk of other STIs as well, instead of using condoms. So I think it makes people more ignorant of the other things as well. And it makes people just more focused on just HIV and not other STIs. And then risk of pregnancy as well is likely to get high, provided the people are not on any other form of contraception.... No, I think... no, from the way you've said it to me... I wouldn't even tell it to people* (HIV-negative African woman)

Most HIV-positive participants described anxieties about PrEP in relation to *their* inability to be in effective control of HIV prevention. One woman could not imagine agreeing to her HIV-negative sexual partner using PrEP: *'[PrEP] is too risky for him because I don't know when he stop using it, what will happen to him.'* (HIV-positive African woman, FG).

Moral barriers to PrEP

For peer review only - http://bmjopen.bmj.com/site/about/guidelines.xhtml

PrEP also emerged as a highly contentious issue because of the perceived negative implications it had for existing risk management strategies and HIV prevention. Many participants viewed PrEP as problematic because they perceived that *others* would stop using condoms if PrEP was available. That is, they distinguished between how *they* might use PrEP and how they perceived others would use it. For some, the concern was in relation to reduced condom use: *'it'll be like... women burning their bras. It'll be all these guys [whipping] off their condoms, do you know what I mean?'* (HIV-negative MSM). Other participants were also concerned with bigger changes in sexual practice, such as increased risk taking:

> *Are you trying to eliminate the condom?... There is no place for a condom at all when it's like this. So I think it actually encourages people to be more promisc... not promiscuous, but to be more... I don't know [if] 'ignorant' is the right word, but to be less careful 'cause they know 'oh, there's that pill'. And it's not even cost-effective than just getting a condom or using...PEP once in a blue moon.* (HIV-negative African woman)

Not all participants were morally opposed to PrEP and many viewed PrEP as a good addition to HIV prevention options. However, there was still concern about *how* PrEP would be accessed. and oOverall it was hoped that it would be made available only under strict conditions to ensure the 'right' people received it and that it was used in the 'correct' way:

> *I believe it should be available in Scotland. But, it should be under strict control to make sure the person who takes it knows that he, or she is not protected to full extent from other infections, and HIV either.* (HIV-negative MSM)

For peer review only - http://bmjopen.bmj.com/site/about/guidelines.xhtml

Discussion

This is the first qualitative study in the UK to report on the acceptability of PrEP~~ amongst potential PrEP users and their HIV-positive sexual partners~~. Our ~~study ~~research offers new insights into the psychological and social barriers to PrEP uptake and use. We identified the ~~importance ~~significance of how the effectiveness of PrEP as a risk reduction intervention is communicated to and understood by potential candidates. We demonstrated the importance of~~HIV literacy, as well as related~~ HIV risk perception and found that, for many participants, PrEP was not immediately seen as a trusted and/or beneficial addition to their repertoire of existing risk reduction practices. Our findings also highlight how existing risk management strategies in relation to PrEP ~~are about more than HIV risk, and ~~encompass broad~~er~~ concerns relating to sexual health, relationships, social factors and communities~~.~~.

Understanding how to interpret PrEP efficacy rates, both on their own and in combination with other prevention strategies, proved a stumbling block for the participants. ~~Although ~~Liu *et al* identify~~y~~ accurate consumer knowledge as~~ a~~ key to PrEP implementation, in addition to addressing other factors such as stigma, adherence, and risk reduction (23)~~(17)~~.~~,~~ While we agree with Liu, ~~o~~our findings suggest that ~~*more* ~~the form and delivery of this~~than ~~ consumer knowledge ~~is needed for~~needs further attention to support ~~informed and ~~effective PrEP use. Negotiating PrEP as a prevention strategy, either on its own ~~and ~~or with other strategies, will require improved levels of HIV literacy to be effective. ~~(~~ We suggest the need to consider *critical* HIV ~~we define HIV l~~literacy skills, which encompass~~ as~~ the ability to know, understand and ~~potentially ~~use~~utilise~~ HIV-related information within existing risk reduction practices(24)~~)~~. ~~Although we did not sample or analyse the data by educational qualification or socio-economic status, o~~Our findings suggest that HIV literacy is directly affected by ~~social inequalities (e.g sexuality, ~~geography, migration status, ~~etc)~~and sero-status and that

For peer review only - http://bmjopen.bmj.com/site/about/guidelines.xhtml

inequalities in HIV literacy within communities affected by HIV , directly affect HIV literacy, and will play an important role in understanding the barriers to PrEP useuptake amongst diverse communities affected by HIV. These factors will have direct implications for both the nature of how PrEP-related HIV risk prevention is delivered and by whom.

For many participants, PrEP was not seen as a necessary or welcome addition to their repertoire of risk management strategies. Our findings suggest that As findings from the VOICE trial attest to, rrisk perception and candidacy will play a critical role in the successfuldecisions to use of PrEP, a finding echoed by Golub *et al* (25) (18). For some of the HIV-negative participants in our study, the rejection of PrEP as unnecessary emerged from a perception that they were not at risk of HIV transmission. HIV risk was managed at a different level, often through the sexual exclusion of HIV-positive sexual partners or through reliance on monogamous sexual relationships. These findings suggest that PrEP implementation strategies will need to engage with these wider, socially embedded risk reduction practices. Moreover, our research demonstrates how the management of risk was not limited only to HIV transmission, but also to risk of STIs, pregnancy and social stigma. Participants described how PrEP would not adequately address existing risks, and had the potential to create significant new risks. Saberi *et al* reported similar moral concerns about the implications of PrEP on condom use in their study with MSM participants in serodiscordant relationships in the US (26)(19). Although they surmise that proximity to HIV and age play a role in these concerns, our findings suggest that moral objections to PrEP were not limited by age or sexuality. We suggest that these moral reactions to PrEP as a risk reduction option are related to broader social and community concerns about the potential for PrEP to radically change the way prevention is practiced (9). This highlights the need to engage with wider social concerns about what constitutes 'inappropriate' or high-risk sexual

For peer review only

practice in relation to PrEP and to demonstrate how PrEP implementation can be a part of a safe and comprehensive risk management strategy.

Our study has a number of strengths and limitations. We employed well-developed and rigorous qualitative methodology which enablesd in-depth exploration of the social meanings of PrEP acceptability and likely use (21)(15). As such, this studyWe therefore adds to existing quantitative PrEP acceptability research. With a small sample of MSM and migrant African participants in a non-generalized HIV epidemic region.from a low HIV prevalence setting, some of whom were engaged in sexual health or community services, our findings are not generalisable to a wider population but we would argue, are transferable to similar populations in similar social contexts. As we did not sample according to sexual risk behaviour, our findings are only transferrable to broad risk groups and not necessarily to 'high risk' individuals. Our inclusion of the recommendation to use condoms in the visual PrEP information may have biased the findings. However, our discussions included consistent and broad descriptions of PrEP and encompassed a wide range of PrEP scenarios, including non-condom use.

Field Code Changed

Our research demonstrates the need to consider acceptability factors which extend beyond drug adherence and risk compensation when introducing and scaling up PrEP and has a number of implications for policy and clinical practice. In particular, it will be necessary to develop clear tools and techniques to communicate PrEP information to potential candidates. These methods will need to translate clinical research relating to PrEP effectiveness in real world contexts as is appropriate in the context of diverse critical literacy skills. Implementation will also need to address low risk perception, non-HIV risk reduction and

For peer review only - http://bmjopen.bmj.com/site/about/guidelines.xhtml

other moral concerns to demonstrate how PrEP can be a part of a safe and comprehensive risk management strategy. Our study suggests that broader social and community concerns about the potential for PrEP to change HIV prevention need to be addressed by supporting the integration of PrEP into existing risk management strategies and through targeted promotion. ~~consider how clinical research concerning effectiveness is translated into real world contexts and communicated to potential users, as well as how potential users are supported in integrating this information into existing risk management strategies. Further research is needed to examine HIV literacy in relation to social inequalities in the understanding of complex trial-based information explicating probabilistic and relative risk. For the effective and efficient implementation of PrEP as a viable prevention strategy, future work is needed to develop clear tools and techniques to communicate PrEP information to potential candidates and to explore the need for the targeted promotion of PrEP.~~

Competing Interests

We have no competing interests.

Author's Contributions

IY designed the study, carried out the qualitative data collection and analysis and drafted the manuscript. PF helped to design the study, carried out some of the data collection and analysis and helped to draft the manuscript. LMD participated in the design of the study and helped to draft the manuscript. All authors read and approved the final manuscript.

Acknowledgements

The HIV and the Biomedical Study, IY and LMD are core funded by the UK Medical Research Council (MRC) (MC_U130031238/MC_UU_12017/2) at the MRC/Chief Scientist Office (CSO) Social and Public Health Sciences Unit, University of Glasgow. PF is funded

For peer review only

by Glasgow Caledonian University. We thank the organisations (Waverly Care, Terrence Higgins Trust Scotland, LGBT Youth Scotland, Gay Men's Health Scotland, Positive Scotland, NHS Greater Glasgow and Clyde, NHS Lothian, NHS Grampian) who helped with recruitment and the men and women who agreed to take part in the focus groups and interviews.

For peer review only

References

1. Baeten JM, Grant R. Use of Antiretrovirals for HIV Prevention: What Do We Know and What Don't We Know? Current HIV/AIDS Reports. 2013;10(2):142-51.

2. Anderson PL, Glidden DV, Liu A, Buchbinder S, Lama JR, Guanira JV, et al. Emtricitabine-Tenofovir Concentrations and Pre-Exposure Prophylaxis Efficacy in Men Who Have Sex with Men. Science Translational Medicine. 2012;4(151):1-8.

3. Young I, McDaid L. How Acceptable are Antiretrovirals for the Prevention of Sexually Transmitted HIV?: A Review of Research on the Acceptability of Oral Pre-exposure Prophylaxis and Treatment as Prevention. AIDS Behav. 2014 Jul 30;18 (2):195-216.

4. Holt M. HIV pre-exposure prophylaxis and treatment as prevention: a review of awareness and acceptability among men who have sex with men in the Asia-Pacific region and the Americas. Sex Health. 2013 Jul 19.

5. Gamarel KE, Golub SA. Intimacy Motivations and Pre-exposure Prophylaxis (PrEP) Adoption Intentions Among HIV-Negative Men Who Have Sex with Men (MSM) in Romantic Relationships. Annals of Behavioral Medicine. 2014:1-10.

6. Kirby T, Thornber-Dunwell M. Uptake of PrEP for HIV slow among MSM. Lancet. 2014;383(9915):399-400.

For peer review only - http://bmjopen.bmj.com/site/about/guidelines.xhtml

7. Amico KR, Stirratt MJ. Adherence to Preexposure Prophylaxis: Current, Emerging, and Anticipated Bases of Evidence. Clinical Infectious Diseases. 2014 July 1, 2014;59(suppl 1):S55-S60.

8. Cairns G. The road to PrEP: trials, regulation and roll-out. aidsmap. 2012.

9. Belluz J. The Truvada wars. BMJ. 2014(348):g3811

10. Kippax S, Stephenson N. Beyond the distinction between biomedical and social dimensions of HIV prevention through the lens of a social public health. Am J Public Health. 2012 May;102(5):789-99.

11. Nguyen VK, Bajos N, Dubois-Arber F, O'Malley J, Pirkle CM. Remedicalizing an epidemic: from HIV treatment as prevention to HIV treatment is prevention. AIDS. 2011 Jan 28;25(3):291-3.

12. Health Protection Scotland. ANSWER: HIV infections and AIDS in Scotland: Quarterly report to 30 June 2013: Health Protection Scotland2013 4 September 2013 Contract No.: 2013/36.

13. PHE. HIV in the United Kingdom: 2013 Report. London: Public Health England2013.

14. Ware NC, Wyatt MA, Haberer JE, Baeten JM, Kintu A, Psaros C, et al. What's love got to do with it? Explaining adherence to oral antiretroviral pre-exposure prophylaxis for HIV-serodiscordant couples. J Acquir Immune Defic Syndr. 2012 Apr 15;59(5):463-8.

15. Grant RM, Lama JR, Anderson PL, McMahan V, Liu AY, Vargas L, et al. Preexposure chemoprophylaxis for HIV prevention in men who have sex with men. New England Journal of Medicine. 2010;363(27):2587-99.

16. Center for Disease Control (CDC). Interim Guidance: Pre-exposure Prophylaxis for the Prevention of HIV Infection in Men Who Have Sex with Men. Morbidity and Mortality Weekly Report. 2011;60(3):65-8.

For peer review only - http://bmjopen.bmj.com/site/about/guidelines.xhtml

17. Cairns G. Consistent condom use in anal sex stops 70% of HIV infections, study finds. 4 March 2013 [10 September 2014]; Available from: http://www.aidsmap.com/Consistent-condom-use-in-anal-sex-stops-70-of-HIV-infections-study-finds/page/2586976/.

18. Weller S, Davis K. Condom effectiveness in reducing heterosexual HIV transmission. Cochrane Database Syst Rev. 2002;1(1).

19. Mason J. Qualitative Researching. London: Sage Publications; 1996.

20. Barbour R, Kitzinger J, editors. Developing Focus Group Research: Politics, Theory and Practice. London: Sage Publications; 1999.

21. Ziebland S, McPherson A. Making sense of qualitative data analysis: an introduction with illustrations from DIPEx (personal experiences of health and illness). Medical Education. 2006;40(5):405-14.

22. Silverman D. Doing Qualitative Research: A Practical Handbook. London: Sage; 2000.

23. Liu A, Cohen S, Follansbee S, Cohan D, Weber S, Sachdev D, et al. Early Experiences Implementing Pre-exposure Prophylaxis (PrEP) for HIV Prevention in San Francisco. PLoS Med. 2014;11(3):e1001613.

24. Sørensen K, Van den Broucke S, Fullam J, Doyle G, Pelikan J, Slonska Z, et al. Health literacy and public health: a systematic review and integration of definitions and models. BMC Public Health. 2012;12(1):80.

25. Golub SA, Gamarel KE, Rendina HJ, Surace A, Lelutiu-Weinberger CL. From efficacy to effectiveness: facilitators and barriers to PrEP acceptability and motivations for adherence among MSM and transgender women in New York City. AIDS patient care and STDs. 2013;27(4):248-54.

Field Code Changed

For peer review only - http://bmjopen.bmj.com/site/about/guidelines.xhtml

26. Saberi P, Gamarel KE, Neilands TB, Comfort M, Sheon N, Darbes LA, et al. Ambiguity, ambivalence, and apprehensions of taking HIV-1 pre-exposure prophylaxis among male couples in San Francisco: a mixed methods study. PLoS One. 2012;7(11):e50061.

For peer review only

Table 1: Focus Group Discussion Guide

Part 1

Participants were presented with a number of objects to discuss. Objects included: condoms, sachets of lubricant, pregnancy test, aidsmap list of ARVs, mocked up bottle of antibiotics, empty boxes/bottles of Truvada, and pictures of: Orasure quicktest and rapid HIV test

- **What do these objects make you think about?**
  - i.e. engagement with sexual health organisations? Own HIV status? Sexual encounters? Pleasure? Community? Privacy?
- **How do these objects relate to risk and HIV?**
  - Who uses these objects? Do you use these objects? Why, or why not?
- **What is risky in relation to HIV?**
  - Is it the person, the sexual position, the location, your own health?
- **Do you use these objects to manage HIV?**
  - If yes, how? If not, why not?
  - Does using these objects hinder/help management of HIV/disclosure of status to others?
- **What else do you use to manage HIV?**
  - i.e. how do you assess risk and how do you manage it? Gaydar profile? Looks?

Part 2

Provide cards of PrEP & TasP provided & explain separately.

- **How might you use these pills?**
  - Ask about serodiscordant partnerships and how these pills might change how HIV transmission is managed?
  - Would the potential use of these pills change depending on: sexual partner, current health experiences, location (space, place), etc?
- **How do you think your friends or sexual partners might use these pills?**

For peer review only - http://bmjopen.bmj.com/site/about/guidelines.xhtml

- Would people choose these pills? Would they choose them in combination with condoms? Would they trust these pills would work? Would this depend on the sexual partner?
- How would you decide to use them? How do you decide on current sexual health practices?

- **Are you concerned about the use of pills as a form of HIV prevention?**
  - Concerns about other STIs/Hep C?
  - Would the side effects be a problem? What are your current experiences with side effects and do you think other men could manage these?

- **Would these pills change the way people currently manage HIV? What do you think about this?**
  - Ask about understandings/experiences of condom use, feelings of security/management of risk with existing options?
  - Would this increase/decrease HIV related stigma?
  - Would this change where the onus of responsibility was placed within the community? (i.e. are HIV positive people more or less responsible to manage transmission now, and would these pills/interventions change things?)

For peer review only - http://bmjopen.bmj.com/site/about/guidelines.xhtml

Tabe 2: Interview Topic Guide for HIV-negative or Untested Participants

1) Experiences with/proximity to HIV

Before I start asking you questions specifically about the latest developments, I'd like to learn a little more about your own experiences with HIV. Can you tell me about that?

Confirm HIV Status: HIV negative/unknown status/undisclosed/other

- Is HIV a risk for you?
  - If yes, what are you concerned about in particular?
  - If you are not concerned about HIV, why not?
  - Has this changed at all for you? (different sexual partners, location, sexual practice, etc)
- Is HIV something that you talk about with your sexual partners? (or friends?)
  - If yes, what do you speak about? In what contexts would you talk about HIV?
  - If not, would you feel able to discuss HIV or any concerns you had about HIV with your sexual partners and/or friends?
- Have you ever tested for HIV?
  - If yes:
    - What prompted you get a test?
    - Where did you get the test done?
    - Have you tested for HIV more than once? How often do you get tested?
  - If not:
    - Have you thought about getting tested before?
    - Is there anything that's stopped you from seeking an HIV test?
    - What might make you get tested?

2) Risk management/sexual health

This study is about understanding how people manage their sexual health and risk of HIV.

- What are the risks for you in relation to sexual health?
- You've said that you manage risk in sexual health by ....;
  - Has this changed over time?
  - Does this change according to sexual partner?
  - Does this change according to location? (home, travelling, public spaces)

For peer review only

- Do you talk to your sexual partners about this?
    - Why/Why not?
- Do you use health or other services to help you manage your sexual health? If so, how?

3) Use of existing technologies –

I'm interested in how people use existing sexual health technologies. This can include lots of things. Here is a list of some of the things that people might use.

List of sexual health technologies presented to participant:

condoms
HIV testing
Sexually transmitted infections (STI) testing
Contraception (the pill, IUD, long-term injections, etc)
pregnancy testing
CD4 counts
viral loads
Anti-retrovirals (ARVs)
post-exposure prophylaxis (PEP)

- Do you use any of these now? Have you used any of these in the past?
    - If not, is there anything that is missing from this list that you or people you know have used?
- How have you used them? What made you use them?
    - How do they work? (explore how is this related to understandings of risk of HIV transmission)
- Have you used any of these in combination with other prevention methods?
- How do you feel about using them?
    - Ease/difficulty of using these?
    - Social concerns?
    - Trust in effectiveness?
- If you started/stopped using some of these, can you say why you did?

4) Potential use of new technologies

For peer review only - http://bmjopen.bmj.com/site/about/guidelines.xhtml

This study is about exploring how people feel about using ARVs, the medication currently used to treat HIV, as a way of preventing HIV. This can be done in a couple of ways.

PrEP

Provide card, but verbally explain:

- *PrEP*– Pre-exposure Prophylaxis (PrEP) is when ARVs are used by people who are HIV negative to prevent the transmission of HIV.
- At the moment, it can be taken once a day, although researchers are looking into other forms (short term PrEP, long-acting injectable, topical gel/microbicides).
- PrEP only works if people take the medication regularly. Clinical trials have shown that it is effective in relation to how often people take the pills.
- People are still encouraged to use condoms and other forms of risk reduction with PrEP use.
- There are some side effects, but this may not affect everyone and it generally seems to be well tolerated in the clinical trials.
- PrEP is not currently available in the UK, but it has been licensed for use in the United States.

- Have you heard of PrEP before?
  - If so, where? From whom? What had you heard?
- What do you think of PrEP as a prevention method?
- How would you feel about using PrEP as a prevention method?
  - If would not, why not?
  - If good, why?
- If you would use PrEP, how do you think you would you use it?
  - In combination with other forms of prevention? As a back-up?
  - In what circumstances might you see PrEP as a potential prevention method?
- Do you have any concerns about PrEP as a prevention method?
  - Concerns about taking medication to prevent HIV transmission? (interactions, remembering tablets, side effects, etc)
  - Do you have any concerns about how it might work?
  - Would there be any social stigma associated with PrEP?
- Do you think other people might use PrEP as a method?
  - What do you think about that?

TasP

For peer review only

Provide card, but verbally explain:

- Treatment as prevention is when ARVs are used by people living with HIV to not only to clinically manage HIV, but also to help prevent the transmission of HIV.
- TasP manages the 'viral load' or the amount of HIV in the system. Research has show that having an 'undetectable' viral load means that transmission of HIV is unlikely to happen. So, if the HIV positive person is taking their treatment regularly, and they don't have an STI, and their viral load is 'undetectable' for a period of time (about 6 months), they would clinically be considered not infectious.
- TasP in particular is when treatment is started to prevent transmission, rather than when the person clinically needs the treatment.
- If someone starts this treatment early – including for prevention reasons – they cannot stop taking this medication. So although people living with HIV will eventually move onto treatment, perhaps after 5 to even 10 years, this would mean starting treatment considerably earlier.

- Have you heard of TasP before?
  - If so, where? From whom? What had you heard?
- What do you think of TasP as a prevention method?
  - What do you think about using drugs to lower levels of infection in people living with HIV?
- Can you imagine using this as a prevention method with a sexual partner who is HIV positive?
  - If yes, why?
  - If not, why not?
- How would you feel if a sexual partner suggested this as an HIV prevention method?
- Do you have any concerns about this as a prevention method?
  - Trust in viral load levels/science?
  - Concerns with social perceptions? (i.e. social stigma around ARVs)
  - Coercion/pressure by sexual partners not to use condoms?
  - Anxieties around STIs?
- How do you think other people who are HIV negative or untested might feel about using ARVs or HIV treatment as a prevention method?
  - What do you think about that?

General themes

In addition to the above themes/areas of enquiry, the interview will pay particular attention to cross-cutting themes and issues such as:

- Gender and sexuality

For peer review only

- Social stigma (HIV-related or otherwise)
- HIV & sexual health literacy
- HIV serostatus as an identity (or not) and how this might function/influence social practices
- Community norms and practices in relation to sexual relations and sexual health
- Social and structural barriers/enablers to sexual health & use of technologies (i.e. access to services, cost, stigma, understandings of rights, legal issues, etc)
- Individual vs group rights and responsibilities
- Trust in science, trust in people

For peer review only - http://bmjopen.bmj.com/site/about/guidelines.xhtml

Figure 1.

PrEP (Pre-Exposure Prophylaxis)

**Who is it for?**	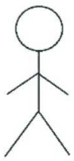 **Person <u>not living with HIV</u> (HIV negative)**
**What is it?**	ARVs are taken **before exposure to HIV** (usually before having sex with someone living with HIV)
**What does this involve?**	PrEP would mostly likely be a pill that would be taken everyday. Side effects could include nausea, diarrhoea, headaches, and tiredness.
**Does this work?**	PrEP is not 100% effective, but **when used with condoms, significantly reduces risk of HIV transmission**.
**Can I get it here?**	No, PrEP is not currently available in Scotland or anywhere else in the UK.

160x225mm (300 x 300 DPI)

Barriers to uptake and use of Pre-Exposure Prophylaxis (PrEP) amongst communities most affected by HIV in the UK: Findings from a qualitative study in Scotland

Journal:	*BMJ Open*
Manuscript ID:	bmjopen-2014-005717.R2
Article Type:	Research
Date Submitted by the Author:	25-Oct-2014
Complete List of Authors:	Young, Ingrid; University of Glasgow, MRC/CSO Social and Public Health Sciences Unit<br>Flowers, Paul; Glasgow Caledonian University, School of Health and Life Sciences<br>McDaid, Lisa; University of Glasgow, MRC/CSO Social and Public Health Sciences Unit
**Primary Subject Heading**:	HIV/AIDS
Secondary Subject Heading:	Qualitative research, Public health, Sexual health, Sociology
Keywords:	HIV & AIDS < INFECTIOUS DISEASES, QUALITATIVE RESEARCH, PUBLIC HEALTH

SCHOLARONE™
Manuscripts

Barriers to uptake and use of Pre-Exposure Prophylaxis (PrEP) amongst communities most affected by HIV in the UK: Findings from a qualitative study in Scotland

Ingrid Young, Paul Flowers, Lisa McDaid

Running Head: Barriers to Pre-Exposure Prophylaxis (PrEP) in the UK

Authors: IYoung[1], P Flowers[2], LM McDaid[1]

Key Words: Pre-Exposure Prophylaxis (PrEP), men who have sex with men (MSM), migrant African communities, Scotland, biomedical HIV prevention

Affiliation:

1. MRC/CSO Social and Public Health Sciences Unit, University of Glasgow
2. School of Health and Life Sciences, Glasgow Caledonian University

Corresponding Author:

Ingrid Young,
MRC/CSO Social and Public Health Sciences Unit, University of Glasgow
200 Renfield Street
Glasgow, G2 3QB
0141 353 7533
ingrid.young@glasgow.ac.uk

For peer review only

Abstract

Objectives: To explore the acceptability of Pre-Exposure Prophylaxis (PrEP) amongst gay, bisexual and men who have sex with men (MSM) and migrant African communities in Scotland, UK.

Design: Consecutive mixed qualitative methods consisting of focus groups (FGs) and in-depth interviews (IDI) explored PrEP acceptability. Data were digitally recorded, transcribed and analysed thematically to identify anticipated and emerging themes.

Setting: Participants were recruited through community sexual health and outreach support services, and from non-sexual health settings across Scotland.

Participants: Inclusion criteria included: identification as either MSM and/or from migrant African communities; 18 years and older; living in Scotland at the time of participation. 7 FGs were conducted (n=33): 5 with MSM (n=22), and 2 mixed-sex groups with African participants (n=11, women=8), aged 18 – 75 years. 34 IDIs were conducted with MSM (n=20) and African participants (n=14, women = 10), aged 19 – 60 years. The sample included participants who were HIV-positive and HIV-negative or untested (HIV-positive FG participants, n=22; HIV-positive IDI participants, n=17).

Results: Understandings of PrEP effectiveness and concerns about maintaining regular adherence were identified as barriers to potential PrEP uptake and use. Low perception of HIV risk due to existing risk management strategies meant few participants saw themselves as PrEP candidates. Participants identified risk of other STIs and pregnancy as a concern which PrEP did not address for either themselves or their sexual partners. PrEP emerged as a contentious issue because of the potentially negative implications it had for HIV prevention. Many participants viewed PrEP as problematic because they perceived that *others* would stop using condoms if PrEP was to become available.

For peer review only - http://bmjopen.bmj.com/site/about/guidelines.xhtml

Conclusions: PrEP implementation needs to: identify appropriate communication methods in the context of diverse HIV literacy; address risk reduction concerns and; demonstrate how PrEP can be part of a safe and comprehensive risk management strategy.

Article summary/Strengths and limitations of this study

- Our study offers new insights into the psychological and social barriers to PrEP uptake and use.
- This paper identifies how limited understandings of PrEP effectiveness and wider community attitudes affect PrEP acceptability.
- We demonstrate the need to consider socially embedded sexual risk management strategies, which address concerns beyond risk of HIV transmission.
- We suggest there is a need to consider how clinical research is translated into real world contexts and communicated to potential users, as well as how potential users are supported in integrating this information into existing risk management strategies.

For peer review only

For peer review only - http://bmjopen.bmj.com/site/about/guidelines.xhtml

Pre-Exposure Prophylaxis (PrEP) represents a significant biomedical addition to international HIV prevention efforts. The clinical efficacy of the use of anti-retrovirals (ARVs) by HIV-negative individuals to prevent the sexual transmission of HIV has been extensively reported (1, 2). The acceptability of PrEP to potential PrEP users (or candidates) to date has focused largely, although not exclusively, on the risk of inefficient PrEP use, such as low or inconsistent adherence to the medication and reduced condom use. It has also focused predominately on experiences in the United States (US) (3-5). There are currently a number of PrEP demonstration studies underway in the United Kingdom (UK), Australia, US and elsewhere to explore how it might be used in 'real world' settings (6, 7). Despite being available in the US since the Food and Drug Administration (FDA) approved Truvada (emtricitabine/tenofovir disoproxil fumarate) for use as PrEP in 2012, there has been relatively low uptake amongst targeted communities affected by HIV, including gay, bisexual and other men who have sex with men (MSM) (6). Moreover, PrEP has emerged as a controversial issue amongst those affected by HIV(8, 9) and there are clear global inequalities in terms of access to PrEP as it is still only available off-label in a number of countries.

There is a need to understand how uptake and use of PrEP as an HIV prevention strategy could be affected by a range of factors, such as community attitudes towards PrEP and the role of PrEP within concurrent HIV prevention strategies of potential PrEP users (10, 11). Here we report on the first qualitative study in the UK of the acceptability of PrEP amongst populations most affected by HIV to inform targeted PrEP implementation strategies .

Methods

This article describes a mixed qualitative methods study on the acceptability of PrEP and Treatment as Prevention (TasP) with two communities in Scotland: 1) MSM and 2) men and

For peer review only - http://bmjopen.bmj.com/site/about/guidelines.xhtml

For peer review only

women from migrant African communities (first generation immigrants including asylum seekers, students and other migrants). HIV is largely concentrated in these two communities UK, as they represent the highest levels of HIV prevalence (12, 13). This article reports on findings in relation to barriers to PrEP use. Our sample included HIV-negative and/or untested participants as potential PrEP users and HIV-positive participants as the potential or existing sexual partners of PrEP users. We include findings from HIV-positive participants because of evidence which indicates that serodiscordant sexual partnerships may be an important factor in PrEP acceptability (14). HIV-positive individuals may shape PrEP uptake through influence on community attitudes, as well as more directly on potential HIV-negative sexual partners.

First, exploratory focus groups (FGs) were conducted with a convenience sample of MSM and African participants between August and November 2012 to identify community attitudes and emerging issues around PrEP acceptability. We conducted seven FGs: five with MSM (n=22), and two mixed sex groups with African participants (n=11). Three FGs were conducted with HIV-positive MSM (n=14) and one FG with HIV-positive Africans (n=8). Participant age ranged between 18 and 75 years and discussions took place in urban and semi-urban locations across central Scotland. Participants were recruited through existing community and/or support groups with the assistance of sexual health and/or LGBT organisations. FG discussion topics included existing risk management strategies in sexual health and an exploration of PrEP and TasP (See Table 1).

We then conducted 34 in-depth interviews (IDIs) between March and September 2013 with a purposive sample of MSM (n=20) and African participants (n=14) to explore issues emerging from FG findings and examine personal risk management practices in further depth. Half of

For peer review only - http://bmjopen.bmj.com/site/about/guidelines.xhtml

the IDI participants were HIV-negative or untested for HIV, and the other half had been diagnosed with HIV at the time of the interview (MSM, n=10; Africans, n=7). Inclusion criteria did not specify risk behaviour to allow for broad exploration of candidacy factors, including those not related to sexual behaviour . IDI participants were aged 19 –60 years and were resident in four Scottish regions (Glasgow, Lothian, Lanarkshire, and Grampian). Tailored flyers and posters were distributed to: workshops and support groups; community sexual health testing clinics; gay bars, saunas and clubs; an MSM mail-out condom programme; commercial venues (e.g. African food and barber shops); and in educational settings known to have large African student populations. Community organisations also made contact with potential participants through their regular online and face-to-face outreach and support work. Interviews took place in private spaces in partner organisations or in participants' own homes, and focused the acceptability of PrEP and TasP, including awareness, potential use, concerns and combination with other and/or existing risk management strategies (such as TasP, serosorting, etc) (See Table 2).

PrEP was explained to participants by drawing on but not limited to the use of a visual aid (Figure 1). Basic explanations of PrEP were consistent across all FG and IDI discussions (Table 2, Section 4a). Subsequent and more detailed descriptions of PrEP varied depending on participant questions, which were encouraged and answered. This approach was taken to identify how PrEP should be described to potential candidates. Material did not specify an exact efficacy rate due to the emerging clinical data, variability according to adherence, and to not overly complicate the explanation. Participants were informed that PrEP efficacy was dependent on levels of adherence, as demonstrated in a number of trials. FG participants were told that the iPrEx study reported approximately 73% protection if taken regularly (90% adherence), which was accurate at the time discussions were conducted (15). IDI participants

For peer review only - http://bmjopen.bmj.com/site/about/guidelines.xhtml

were informed that efficacy could be up to or more than 90% if taken regularly, drawing on subsequent sub-analyses of clinical findings (2). Participants were informed that other forms of risk reduction were recommended, such as condoms (16) . Efficacy of condoms was described as less than 100% (17, 18). Discussions explored a wide-range of PrEP scenarios, including non-condom use.

Written consent was provided by all participants at the start of the FGs and IDIs. All FGs and IDIs were digitally recorded and transcribed verbatim. Transcripts were anonymised and coded in NVivo 10. Data were analysed thematically, drawing on both anticipated and emergent themes (19-21). Rigour throughout the analysis was achieved through an iterative process of discussion and revision between co-authors (19, 20, 22). Ethical approval was obtained from the University of Glasgow's College of Social Sciences Ethics Committee (CSS2012/0193, CSS20120264).

Results

We identified five potential barriers to effective PrEP use: interpreting effectiveness; managing adherence; PrEP candidacy and low perceptions of HIV risk; concerns with other risks such as the criminalization of HIV transmission and STIs; and moral barriers. We have identified extracts taken from FGs; otherwise, it can be assumed that the extract comes from an IDI participant.

Interpreting effectiveness

Understandings of PrEP effectiveness emerged as an important barrier to potential and effective use. Although participants were informed that PrEP was highly effective when

For peer review only

taken regularly, most participants expressed concerns that it provided less than 100% protection and therefore was 'insufficient' to prevent HIV transmission on its own. Some participants felt that PrEP used in isolation was 'too much' of a risk (if only approximately 70% effective).

> ***Respondent 1****: That's not enough, that's not enough, exactly.*
>
> ***Respondent 2:*** *Its seventy-two per cent effective but then there's still that twenty-eight per cent.*
>
> (HIV-Negative MSM, FG)

Participants also expressed a wider scepticism of PrEP, which emerged in two ways. Some thought that the advice to use condoms with PrEP indicated a continued scientific uncertainty: '*I don't know if this [will] work because since they said if you use it you can still use condom, that means they are not sure as well'* (HIV-negative African woman). Second, some expressed scepticism in relation to the variability in reported efficacy rates in relation to adherence. As a result, there was a reluctance to trust these, unless the information came from a recognised, trusted and reliable source:

> ***R1:*** *There's so much out there right, on the internet an' everything else right, if it came up on the news right? The logistic news on BBC one that this pill will prevent so…—like a hundred per cent – that's when I would believe it would work. I [would not] trust any other pill ...*
>
> ***Question:*** *So it has to be a hundred per cent?*
>
> ***R2:*** *'cause there's a lot a... no like—anyone can put something on the internet,... You've got to use like certain websites, you've got to look like .org, .gov, those type o' websites. You've just done one that's like www.medicine.co.uk it's not a... it's not like a legitimate website it could just be anyone postin' stuff up there.*

For peer review only - http://bmjopen.bmj.com/site/about/guidelines.xhtml

Q: *Well I suppose in the States it's from the Food and Drug Administration so it's got that stamp on it.*

R1*: Yes, but you [don't know] if they're been a hundred per cent accurate on their job or just been lazy an' the fact that we're brave enough to take it to human trials right?*

(HIV-Negative MSM, FG)

In spite of being told that the FDA had approved the drug, these FG participants did not recognise the organisation or trust it as a reliable source, and were therefore sceptical about its statements and/or endorsement.

Other participants were less concerned about PrEP not being 100% efficacious because they imagined that it would be used *in addition* to condoms. However, participants expressed confusion or uncertainty in knowing how to interpret efficacy rates and their impact on risk reduction in this particular context. For example, in discussing the effectiveness of condoms in relation to other prevention strategies, one participant calculated the protection offered by PrEP, condoms and TasP with a hypothetical HIV-positive sexual partner. Assuming that PrEP was 90% effective at reducing HIV transmission, he explained:

R*: but there's obviously still a 10% risk but, as you said, there's the same risk with condoms. So it's either you take the 10% risk or you say 'well, I'll use condoms and we'll use TASP' which makes 180%. (Laugh)*

Q*: And PrEP. (Laugh)*

R*: 253 out of 300!*

(HIV-negative MSM)

For peer review only - http://bmjopen.bmj.com/site/about/guidelines.xhtml

Although the participant in this extract was joking about adding up the numbers, the interpretation of efficacy rates in relation to other prevention options posed a potential source of confusion or misinformation for participants.

Managing adherence

Given the evidence concerning the patterning of efficacy by adherence (2), maintaining regular adherence to medication was identified as a potential barrier to effective PrEP use. Some participants described how they might forget to take tablets or their routine might be disrupted because of non-regular working patterns, and were therefore concerned about how effective PrEP would be if they did not take the drugs regularly: '*Sometimes I forget. Because I don't have a regular sleeping pattern, so sometimes I'll fall asleep at four in the afternoon and I usually take my pills [for an existing health condition] after dinner'* (HIV-Negative MSM). Establishing and maintaining a routine to take PrEP was also affected by the perception of social stigma attached to HIV medication. This meant maintaining daily adherence would be difficult if there was limited privacy (e.g. the presence of room-mates) or if there was a change in environment. One participant reported concerns about his family potentially finding the tablets:

> *...if I was on those medications for a year and if I went home...or anything like that I'd find it very difficult to be able to take my medications or I would find it a bit of a barrier that if my family knew about it they'd investigate why are you on these pills. And again that would probably put some doubt in their head and then they'd probably then think the worst - that I was HIV positive.*
>
> (HIV-negative MSM)

For peer review only - http://bmjopen.bmj.com/site/about/guidelines.xhtml

In addition to being unable to establish or maintain a regular routine to facilitate good adherence, most participants expressed concerns about the physical effects of the drugs themselves, and how the side-effects might inhibit taking them. Some participants described disliking taking tablets and that this would be a barrier to daily PrEP use: *I don't really like pills so I don't think I would take any pills every day'* (HIV-negative African man). Many viewed the potential for side effects, such as nausea and diarrhoea, as too great a trade-off for increased HIV prevention, in spite of being informed that trials had reported high tolerance of the drugs:

> *But, could I put up wi' side-effects all the time as well, like taking a pill, and going tae a nightclub, and ended up wi' diarrhoea? You know, you've gotta think of things like that, am I [going to] take this? Am I [going to] stand tired, have a headache in a club just at the fact that I might at the end of it get a shag...*(HIV-Negative MSM)

Other participants felt the side-effects would interfere with sexual practice itself: '*Nausea, an' diarrhoea an'—you'll be shittin' all over his [penis]. No [thanks].'* (HIV-negative MSM, FG) A large minority of both HIV-positive and HIV-negative participants raised the issue of longer-term side-effects, especially those who were more familiar with the effects of ARVs:

> *Does it not have any side effects inside my body? 'Cause from my reading as well, some of these anti-retrovirals have got side-effects with the lungs, with the kidneys and stuff. So, in the long run, if I get used to drinking this pill and I'm actually not exposed because I'm thinking I might be at risk of getting exposed, am I not doing more damage to myself in my body?* (HIV-Negative African woman)

PrEP candidacy and low perceptions of HIV risk

Many participants described scepticism in taking PrEP daily, especially if they were not

For peer review only - http://bmjopen.bmj.com/site/about/guidelines.xhtml

always exposed to or at risk of HIV transmission. As such, perceptions of HIV transmission risk played an important role in potential uptake of PrEP. Several participants rejected the use of PrEP because they perceived themselves to be at a very low risk. For the majority of HIV-negative participants, this was because most adopted serosorting or believed they could accurately tell if a potential partner was HIV-positive and avoid sex with him or her: '*I wouldn't just [want to] take a risk because it's already defining as a risk, I mean, it's a risk, it's already been defined as a risk and since I'm a little bit risk averse... I won't just do anything'* (HIV-negative African man). HIV stigma and assumptions about disclosure appeared to inform these strategies. For example, some HIV-negative participants assumed that HIV-disclosure to sexual partners '*was the law'* (HIV-negative MSM, FG), and that their sexual partners were not HIV-positive. Those HIV-negative participants in sero-discordant relationships and HIV-positive participants suggested that condoms, and the nature of their relationships (e.g. monogamous), already helped manage their risks, and that the additional benefit from PrEP was unnecessary. One man in a sero-discordant relationship explained:

> *Right now in my current situation as, I'm doing a monogamous relationship and everything like that, again I don't think even in that [instance] I don't think I would take the pills even though it would be an extra measure...I think I would feel comfortable enough in the current situation.* (HIV-Negative MSM)

PrEP and concerns with other risks

Both HIV-positive and HIV-negative participants identified risk of other sexually-transmitted infections (STIs) as a concern for both themselves and their sexual partners. Heterosexual African participants, especially African women, also talked of the risk of pregnancy. For those participants who were either living with HIV, or who had experience of sero-discordant

For peer review only - http://bmjopen.bmj.com/site/about/guidelines.xhtml

relationships, other health risks posed to the HIV-positive sexual partner through PrEP use were also identified:

> *when I'm looking at condom use it's not just HIV that you're protecting yourself against but some other STIs as well. So you're killing two birds with one stone, more like it. But when it's now using this, you're actually getting yourself involved in something that you already know – 'this person is positive and I'm [going to] have sex with him, but it's okay, I will not use condoms because I know I can use this.' But you're also putting that person, because that person's immune system might be weak. They're also putting that person at risk of other STIs as well, instead of using condoms. So I think it makes people more ignorant of the other things as well. And it makes people just more focused on just HIV and not other STIs. And then risk of pregnancy as well is likely to get high, provided the people are not on any other form of contraception.... No, I think... no, from the way you've said it to me... I wouldn't even tell it to people* (HIV-negative African woman)

Most HIV-positive participants described anxieties about PrEP in relation to *their* inability to be in effective control of HIV prevention. One woman could not imagine agreeing to her HIV-negative sexual partner using PrEP: *'[PrEP] is too risky for him because I don't know when he stop using it, what will happen to him'* (HIV-positive African woman, FG).

Moral barriers to PrEP

PrEP emerged as a highly contentious issue because of the perceived negative implications it had for existing risk management strategies and HIV prevention. Many participants viewed PrEP as problematic because they perceived that *others* would stop using condoms if PrEP was available. That is, they distinguished between how *they* might use PrEP and how they

For peer review only - http://bmjopen.bmj.com/site/about/guidelines.xhtml

perceived others would use it. For some, the concern was in relation to reduced condom use: '*it'll be like... women burning their bras. It'll be all these guys [whipping] off their condoms, do you know what I mean?'* (HIV-negative MSM). Other participants were also concerned with bigger changes in sexual practice, such as increased risk taking:

> *Are you trying to eliminate the condom?... There is no place for a condom at all when it's like this. So I think it actually encourages people to be more promisc... not promiscuous, but to be more... I don't know [if] 'ignorant' is the right word, but to be less careful 'cause they know 'oh, there's that pill'. And it's not even cost-effective than just getting a condom or using...PEP once in a blue moon.* (HIV-negative African woman)

Not all participants were opposed to PrEP and many viewed PrEP as a good addition to HIV prevention options. However, there was still concern about *how* PrEP would be accessed. Overall it was hoped that it would be made available only under strict conditions to ensure the 'right' people received it and that it was used in the 'correct' way:

> *I believe it should be available in Scotland. But, it should be under strict control to make sure the person who takes it knows that he, or she is not protected to full extent from other infections, and HIV either.* (HIV-negative MSM)

Discussion

This is the first qualitative study in the UK to report on the acceptability of PrEP. Our research offers new insights into the psychological and social barriers to PrEP uptake and use. We identified the significance of how the effectiveness of PrEP as a risk reduction intervention is communicated to and understood by potential candidates. Our research

For peer review only - http://bmjopen.bmj.com/site/about/guidelines.xhtml

suggests the importance of HIV risk perception and found that, for many participants, PrEP was not immediately seen as a trusted and/or beneficial addition to their repertoire of existing risk reduction practices. Our findings also highlight how existing risk management strategies in relation to PrEP encompass broad concerns relating to sexual health, relationships, social factors and communities.

Understanding how to interpret PrEP efficacy rates, both on their own and in combination with other prevention strategies, proved a stumbling block for the participants and poses a considerable challenge to how health providers support the concept of combination prevention in the context of PrEP. Liu *et al* identify accurate consumer knowledge as key to PrEP implementation, in addition to addressing other factors such as stigma, adherence, and risk reduction (23). While we agree with Liu, our findings suggest that the form and delivery of this consumer knowledge, including *how* health providers understand and communicate this information, needs further attention to support effective PrEP use. Communicating PrEP effectiveness in real world settings will be a two-way process that demands clarity on the part of both providers and potential users. In addition to supporting providers, negotiating PrEP as a prevention strategy will require improved levels of HIV literacy amongst potential PrEP users to be effective. We suggest the need to consider *critical* HIV literacy, which encompasses the ability to know, understand and use HIV-related information within existing risk reduction practices (24). As levels of HIV knowledge are directly affected by a range of factors, such as proximity to HIV(25), inequalities in HIV literacy within communities affected by HIV will play an important role in understanding the barriers to PrEP use. These factors have direct implications for both the nature of how PrEP-related HIV risk prevention is delivered and by whom.

For peer review only

For peer review only - http://bmjopen.bmj.com/site/about/guidelines.xhtml

For many participants, PrEP was not seen as a necessary or welcome addition to their repertoire of risk management strategies. Our findings suggest that risk perception and candidacy will play a critical role in decisions to use PrEP, a finding echoed by Golub *et al* (26). For some of the HIV-negative and/or untested participants in our study, the rejection of PrEP as unnecessary emerged from a perception that they were not at risk of HIV transmission. HIV risk was managed often through the sexual exclusion of HIV-positive sexual partners or through reliance on monogamous sexual relationships. These findings suggest that PrEP implementation strategies will need to engage with these wider, socially embedded risk reduction practices, including how HIV stigma might affect risk perception. Moreover, our research highlights how the management of risk was not limited only to HIV transmission, but also to risk of STIs, pregnancy and social stigma. Participants described how PrEP would not adequately address existing risks, and had the potential to create significant new risks. Saberi *et al* reported similar moral concerns about the implications of PrEP on condom use in their study with MSM participants in serodiscordant relationships in the US (27). Although they surmise that proximity to HIV and age play a role in these concerns, our findings suggest that moral objections to PrEP were not limited by age or sexuality. We suggest that these moral reactions to PrEP as a risk reduction option are related to broader social and community concerns about the potential for PrEP to radically change the way prevention is practiced (9). This highlights the need to engage with wider social concerns about what constitutes 'inappropriate' or high-risk sexual practice in relation to PrEP and to demonstrate how PrEP implementation can be a part of a safe and comprehensive risk management strategy.

Our study has a number of strengths and limitations. We employed rigorous qualitative methodology which enabled in-depth exploration of the social meanings of PrEP

For peer review only - http://bmjopen.bmj.com/site/about/guidelines.xhtml

acceptability and likely use (21). We therefore add to existing quantitative PrEP acceptability research. With a small sample of MSM and migrant African participants in a non-generalized HIV epidemic, some of whom were engaged in sexual health or community services, our findings are not generalisable to a wider population but we would argue, are transferable to similar populations in similar social contexts. As we did not sample according to sexual risk behaviour, our findings are only transferrable to broad risk groups and not necessarily to 'high risk' individuals. Our inclusion of the recommendation to use condoms in the visual PrEP information may have biased the findings. However, our discussions included consistent and broad descriptions of PrEP and encompassed a wide range of PrEP scenarios, including non-condom use.

Our research identifies the need to consider acceptability factors which extend beyond drug adherence and risk compensation when introducing and scaling up PrEP and has a number of implications for policy and clinical practice. In particular, it will be necessary to develop clear tools and techniques to communicate PrEP information to potential candidates, and to support health providers in the implementation of these tools. These methods will need to translate clinical research relating to PrEP effectiveness in real world contexts as is appropriate in the context of diverse critical literacy skills. Implementation will also need to address low risk perception, non-HIV related risk reduction and other moral concerns to demonstrate how PrEP can be a part of a safe and comprehensive risk management strategy. Our study suggests that broader social and community concerns about the potential for PrEP to change HIV prevention need to be addressed by supporting the integration of PrEP into existing risk management strategies and through targeted promotion.

For peer review only - http://bmjopen.bmj.com/site/about/guidelines.xhtml

For peer review only

Competing Interests

We have no competing interests.

Author's Contributions

IY designed the study, carried out the qualitative data collection and analysis and drafted the manuscript. PF helped to design the study, carried out some of the data collection and analysis and helped to draft the manuscript. LMD participated in the design of the study and helped to draft the manuscript. All authors read and approved the final manuscript.

Acknowledgements

The HIV and the Biomedical Study, IY and LMD are core funded by the UK Medical Research Council (MRC) (MC_U130031238/MC_UU_12017/2) at the MRC/Chief Scientist Office (CSO) Social and Public Health Sciences Unit, University of Glasgow. PF is funded by Glasgow Caledonian University. We thank the organisations (Waverly Care, Terrence Higgins Trust Scotland, LGBT Youth Scotland, Gay Men's Health Scotland, Positive Scotland, NHS Greater Glasgow and Clyde, NHS Lothian, NHS Grampian) who helped with recruitment and the men and women who agreed to take part in the focus groups and interviews.

References

1. Baeten JM, Grant R. Use of Antiretrovirals for HIV Prevention: What Do We Know and What Don't We Know? Current HIV/AIDS Reports. 2013;10(2):142-51.

2. Anderson PL, Glidden DV, Liu A, Buchbinder S, Lama JR, Guanira JV, et al. Emtricitabine-Tenofovir Concentrations and Pre-Exposure Prophylaxis Efficacy in Men Who Have Sex with Men. Science Translational Medicine. 2012;4(151):1-8.

For peer review only - http://bmjopen.bmj.com/site/about/guidelines.xhtml

3. Young I, McDaid L. How Acceptable are Antiretrovirals for the Prevention of Sexually Transmitted HIV?: A Review of Research on the Acceptability of Oral Pre-exposure Prophylaxis and Treatment as Prevention. AIDS Behav. 2014 Jul 30;18 (2):195-216.

4. Holt M. HIV pre-exposure prophylaxis and treatment as prevention: a review of awareness and acceptability among men who have sex with men in the Asia-Pacific region and the Americas. Sex Health. 2013 Jul 19.

5. Gamarel KE, Golub SA. Intimacy Motivations and Pre-exposure Prophylaxis (PrEP) Adoption Intentions Among HIV-Negative Men Who Have Sex with Men (MSM) in Romantic Relationships. Annals of Behavioral Medicine. 2014:1-10.

6. Kirby T, Thornber-Dunwell M. Uptake of PrEP for HIV slow among MSM. Lancet. 2014;383(9915):399-400.

7. Amico KR, Stirratt MJ. Adherence to Preexposure Prophylaxis: Current, Emerging, and Anticipated Bases of Evidence. Clinical Infectious Diseases. 2014 July 1, 2014;59(suppl 1):S55-S60.

8. Cairns G. The road to PrEP: trials, regulation and roll-out. aidsmap. 2012.

9. Belluz J. The Truvada wars. BMJ. 2014(348):g3811

10. Kippax S, Stephenson N. Beyond the distinction between biomedical and social dimensions of HIV prevention through the lens of a social public health. Am J Public Health. 2012 May;102(5):789-99.

11. Nguyen VK, Bajos N, Dubois-Arber F, O'Malley J, Pirkle CM. Remedicalizing an epidemic: from HIV treatment as prevention to HIV treatment is prevention. AIDS. 2011 Jan 28;25(3):291-3.

12. Health Protection Scotland. ANSWER: HIV infections and AIDS in Scotland: Quarterly report to 30 June 2013: Health Protection Scotland2013 4 September 2013 Contract No.: 2013/36.

For peer review only

For peer review only - http://bmjopen.bmj.com/site/about/guidelines.xhtml

13. PHE. HIV in the United Kingdom: 2013 Report. London: Public Health England2013.

14. Ware NC, Wyatt MA, Haberer JE, Baeten JM, Kintu A, Psaros C, et al. What's love got to do with it? Explaining adherence to oral antiretroviral pre-exposure prophylaxis for HIV-serodiscordant couples. J Acquir Immune Defic Syndr. 2012 Apr 15;59(5):463-8.

15. Grant RM, Lama JR, Anderson PL, McMahan V, Liu AY, Vargas L, et al. Preexposure chemoprophylaxis for HIV prevention in men who have sex with men. New England Journal of Medicine. 2010;363(27):2587-99.

16. Center for Disease Control (CDC). Interim Guidance: Pre-exposure Prophylaxis for the Prevention of HIV Infection in Men Who Have Sex with Men. Morbidity and Mortality Weekly Report. 2011;60(3):65-8.

17. Cairns G. Consistent condom use in anal sex stops 70% of HIV infections, study finds. 4 March 2013 [10 September 2014]; Available from: http://www.aidsmap.com/Consistent-condom-use-in-anal-sex-stops-70-of-HIV-infections-study-finds/page/2586976/.

18. Weller S, Davis K. Condom effectiveness in reducing heterosexual HIV transmission. Cochrane Database Syst Rev. 2002;1(1).

19. Mason J. Qualitative Researching. London: Sage Publications; 1996.

20. Barbour R, Kitzinger J, editors. Developing Focus Group Research: Politics, Theory and Practice. London: Sage Publications; 1999.

21. Ziebland S, McPherson A. Making sense of qualitative data analysis: an introduction with illustrations from DIPEx (personal experiences of health and illness). Medical Education. 2006;40(5):405-14.

22. Silverman D. Doing Qualitative Research: A Practical Handbook. London: Sage; 2000.

For peer review only

For peer review only - http://bmjopen.bmj.com/site/about/guidelines.xhtml

23. Liu A, Cohen S, Follansbee S, Cohan D, Weber S, Sachdev D, et al. Early Experiences Implementing Pre-exposure Prophylaxis (PrEP) for HIV Prevention in San Francisco. PLoS Med. 2014;11(3):e1001613.

24. Sørensen K, Van den Broucke S, Fullam J, Doyle G, Pelikan J, Slonska Z, et al. Health literacy and public health: a systematic review and integration of definitions and models. BMC Public Health. 2012;12(1):80.

25. Keogh P. Morality, responsibility and risk: Negative gay men's perceived proximity to HIV. AIDS Care. 2008;20(5):576-81.

26. Golub SA, Gamarel KE, Rendina HJ, Surace A, Lelutiu-Weinberger CL. From efficacy to effectiveness: facilitators and barriers to PrEP acceptability and motivations for adherence among MSM and transgender women in New York City. AIDS patient care and STDs. 2013;27(4):248-54.

27. Saberi P, Gamarel KE, Neilands TB, Comfort M, Sheon N, Darbes LA, et al. Ambiguity, ambivalence, and apprehensions of taking HIV-1 pre-exposure prophylaxis among male couples in San Francisco: a mixed methods study. PLoS One. 2012;7(11):e50061.

For peer review only

Table 1: Focus Group Discussion Guide

Part 1

Participants were presented with a number of objects to discuss. Objects included: condoms, sachets of lubricant, pregnancy test, list of ARVs, mocked up bottle of antibiotics, empty boxes/bottles of Truvada, and pictures of: Orasure quicktest and rapid HIV test

- **What do these objects make you think about?**
- **How do these objects relate to risk and HIV?**
- **What is risky in relation to HIV?**
- **Do you use these objects to manage HIV?**
- **What else do you use to manage HIV?**

Part 2

Provide visual cards of PrEP & TasP provided & explain separately.

- **How might you use these pills?**
- **How do you think your friends or sexual partners might use these pills?**
- **Are you concerned about the use of pills as a form of HIV prevention?**
- **Would these pills change the way people currently manage HIV? What do you think about this?**

For peer review only

Table 2: Interview Topic Guide for HIV-negative or Untested Participants

1) Experiences with and/or proximity to HIV

- Is HIV a risk for you?
- Is HIV something that you talk about with your sexual partners? (or friends?)
- Have you ever tested for HIV?

2) Risk management/sexual health

- What are the risks for you in relation to sexual health?
- You've said that you manage risk in sexual health by ..... Has this changed and how?
- Do you talk to your sexual partners about how you manage your sexual health?
- Do you use health or other services to help you manage your sexual health? If so, how?

3) Use of existing technologies –

List of sexual health technologies physically presented to participant:

condoms
HIV testing
Sexually transmitted infections (STI) testing
Contraception (the pill, IUD, long-term injections, etc)
pregnancy testing
CD4 counts
viral loads
Anti-retrovirals (ARVs)
post-exposure prophylaxis (PEP)

- Do you use any of these now? Have you used any of these in the past?
- How have you used them? What made you use them?
- Have you used any of these in combination with other prevention methods?
- How do you feel about using them?
- If you started/stopped using some of these, can you say why you did?

4) Potential use of new technologies

a) PrEP

Approximate PrEP description explained to participant:

Pre-exposure Prophylaxis (PrEP) is when ARVs are used by people who are HIV negative to prevent the transmission of HIV. At the moment, it can be taken once a day, although researchers are looking into other forms (short term PrEP, long-acting injectable, topical gel/microbicides). PrEP only works if people take the medication regularly. Clinical trials have shown that it is effective in relation to how often people take the pills. People are still encouraged to use condoms and other forms of risk reduction with PrEP use. There are some side effects, but this may not affect everyone and it generally seems to be well tolerated in the clinical trials. PrEP is not currently available in the UK, but it has been licensed for use in the United States.

For peer review only

For peer review only - http://bmjopen.bmj.com/site/about/guidelines.xhtml

- Have you heard of PrEP before?
- What do you think of PrEP as a prevention method?
- How would you feel about using PrEP as a prevention method?
- If you would use PrEP, how do you think you would you use it?
- Do you have any concerns about PrEP as a prevention method?
- Do you think other people might use PrEP as a method?

b) TasP

Approximate TasP description explained to participant:

Treatment as prevention is when ARVs are used by people living with HIV to not only to clinically manage HIV, but also to help prevent the transmission of HIV. TasP manages the 'viral load' or the amount of HIV in the system. Research has show that having an 'undetectable' viral load means that transmission of HIV is unlikely to happen. So, if the HIV positive person is taking their treatment regularly, and they don't have an STI, and their viral load is 'undetectable' for a period of time (about 6 months), they would clinically be considered not infectious. TasP in particular is when treatment is started to prevent transmission, rather than when the person clinically needs the treatment. If someone starts this treatment early – including for prevention reasons – they cannot stop taking this medication. So although people living with HIV will eventually move onto treatment, perhaps after 5 to even 10 years, this would mean starting treatment considerably earlier.

- Have you heard of TasP before?
- What do you think of TasP as a prevention method?
- Can you imagine using this as a prevention method with a sexual partner who is HIV positive?
- How would you feel if a sexual partner suggested this as an HIV prevention method?
- Do you have any concerns about this as a prevention method?
- How do you think other people who are HIV negative or untested might feel about using ARVs or HIV treatment as a prevention method?

For peer review only

For peer review only - http://bmjopen.bmj.com/site/about/guidelines.xhtml

Barriers to uptake and use of Pre-Exposure Prophylaxis (PrEP) amongst communities most affected by HIV in the UK: Findings from a qualitative study in Scotland

Ingrid Young, Paul Flowers, Lisa McDaid

Running Head: Barriers to Pre-Exposure Prophylaxis (PrEP) in the UK

Authors: IYoung[1], P Flowers[2], LM McDaid[1]

Key Words: Pre-Exposure Prophylaxis (PrEP), men who have sex with men (MSM), migrant African communities, Scotland, biomedical HIV prevention

Affiliation:

1. MRC/CSO Social and Public Health Sciences Unit, University of Glasgow
2. School of Health and Life Sciences, Glasgow Caledonian University

Corresponding Author:

Ingrid Young,
MRC/CSO Social and Public Health Sciences Unit, University of Glasgow
200 Renfield Street
Glasgow, G2 3QB
0141 353 7533
ingrid.young@glasgow.ac.uk

For peer review only

For peer review only - http://bmjopen.bmj.com/site/about/guidelines.xhtml

Abstract

Objectives: To explore the acceptability of Pre-Exposure Prophylaxis (PrEP) amongst gay, bisexual and men who have sex with men (MSM) and migrant African communities in Scotland, UK.

Design: Consecutive mixed qualitative methods consisting of focus groups (FGs) and in-depth interviews (IDI) explored PrEP acceptability. Data were digitally recorded, transcribed and analysed thematically to identify anticipated and emerging themes.

Setting: Participants were recruited through community sexual health and outreach support services, and from non-sexual health settings across Scotland.

Participants: Inclusion criteria included: identification as either MSM and/or from migrant African communities; 18 years and older; living in Scotland at the time of participation. 7 FGs were conducted (n=33): 5 with MSM (n=22), and 2 mixed-sex groups with African participants (n=11, women=8), aged 18 – 75 years. 34 IDIs were conducted with MSM (n=20) and African participants (n=14, women = 10), aged 19 – 60 years. The sample included participants who were HIV-positive and HIV-negative or untested (HIV-positive FG participants, n=22; HIV-positive IDI participants, n=17).

Results: Understandings of PrEP effectiveness and concerns about maintaining regular adherence were identified as barriers to potential PrEP uptake and use. Low perception of HIV risk due to existing risk management strategies meant few participants saw themselves as PrEP candidates. Participants identified risk of other STIs and pregnancy as a concern which PrEP did not address for either themselves or their sexual partners. PrEP emerged as a contentious issue because of the potentially negative implications it had for HIV prevention. Many participants viewed PrEP as problematic because they perceived that *others* would stop using condoms if PrEP was to become available.

For peer review only

Conclusions: PrEP implementation needs to: identify appropriate communication methods in the context of diverse HIV literacy; address risk reduction concerns and; demonstrate how PrEP can be part of a safe and comprehensive risk management strategy.

Article summary/Strengths and limitations of this study

- Our study offers new insights into the psychological and social barriers to PrEP uptake and use.
- This paper identifies how ~~inequalities in critical HIV literacy~~ limited understandings of PrEP effectiveness and wider community attitudes affect PrEP acceptability.
- We demonstrate the need to consider socially embedded sexual risk management strategies, which address concerns beyond risk of HIV transmission.
- We suggest there is a need to consider how clinical research is translated into real world contexts and communicated to potential users, as well as how potential users are supported in integrating this information into existing risk management strategies.

For peer review only

For peer review only - http://bmjopen.bmj.com/site/about/guidelines.xhtml

Pre-Exposure Prophylaxis (PrEP) represents a significant biomedical addition to international HIV prevention efforts. The clinical efficacy of the use of anti-retrovirals (ARVs) by HIV-negative individuals to prevent the sexual transmission of HIV has been extensively reported (1, 2). The acceptability of PrEP to potential PrEP users (or candidates) to date has focused largely, although not exclusively, on the risk of inefficient PrEP use, such as low or inconsistent adherence to the medication and reduced condom use. It has also focused predominately on experiences in the United States (US) (3-5). There are currently a number of PrEP demonstration studies underway in the United Kingdom (UK), Australia, US and elsewhere to explore how it might be used in 'real world' settings (6, 7). Despite being available in the US since the Food and Drug Administration (FDA) approved Truvada (emtricitabine/tenofovir disoproxil fumarate) for use as PrEP in 2012, there has been relatively low uptake amongst targeted communities affected by HIV, including gay, bisexual and other men who have sex with men (MSM) (6). Moreover, PrEP has emerged as a controversial issue amongst those affected by HIV(8, 9) and there are clear global inequalities in terms of access to PrEP as it is still only available off-label in a number of countries..

There is a need to understand how uptake and use of PrEP as an HIV prevention strategy could be affected by a range of factors, such as community attitudes towards PrEP and the role of PrEP within concurrent HIV prevention strategies of potential PrEP users (10, 11). Here we report on the first qualitative study in the UK of the acceptability of PrEP amongst populations most affected by HIV to inform targeted PrEP implementation strategies. .

Methods

This article describes a mixed qualitative methods study on the acceptability of PrEP and Treatment as Prevention (TasP) with two communities in Scotland: 1) MSM and 2) men and

For peer review only - http://bmjopen.bmj.com/site/about/guidelines.xhtml

women from migrant African communities (first generation immigrants including asylum seekers, students and other migrants). HIV is largely concentrated in these two communities UK, as they represent the highest levels of HIV prevalence (12, 13). This article reports on findings in relation to barriers to PrEP use. Our sample included HIV-negative and/or untested participants as potential PrEP users and HIV-positive participants as the potential or existing sexual partners of PrEP users. We include findings from HIV-positive participants because of evidence which indicates that serodiscordant sexual partnerships may be an important factor in PrEP acceptability (14). HIV-positive individuals may shape PrEP uptake through influence on -community attitudes, as well as more directly on potential HIV-negative sexual partners.. We did not explicitly work with serodiscordant couples so as not to exclude potential acceptability factors and to gather wider community attitudes towards PrEP.

First, exploratory focus groups (FGs) were conducted with a convenience sample of MSM and African participants between August and November 2012 to identify community attitudes and emerging issues around PrEP acceptability. We conducted seven FGs: five with MSM (n=22), and two mixed sex groups with African participants (n=11). Three FGs were conducted with HIV-positive MSM (n=14) and one FG with HIV-positive Africans (n=8). Participant age ranged between 18 and 75 years and discussions took place in urban and semi-urban locations across central Scotland. Participants were recruited through existing community and/or support groups with the assistance of sexual health and/or LGBT organisations. FG discussion topics included existing risk management strategies in sexual health, use of sexual health technologies and an exploration of PrEP and TasP (See Table 1).

We then conducted 34 in-depth interviews (IDIs) between March and September 2013 with a purposive sample of MSM (n=20) and African participants (n=14) to explore issues emerging

For peer review only - http://bmjopen.bmj.com/site/about/guidelines.xhtml

from FG findings and examine personal risk management practices in further depth. Half of the IDI participants were HIV-negative or untested for HIV, and the other half had been diagnosed with HIV at the time of the interview (MSM, n=10; Africans, n=7). Inclusion criteria did not specify risk behaviour to allow for findings in relation to broad exploration of candidacy factors, including those not related to sexual behaviour -. IDI participants were aged 19 –60 years and were resident in four Scottish regions (Glasgow, Lothian, Lanarkshire, and Grampian). Tailored flyers and posters were distributed to: workshops and support groups; community sexual health testing clinics; gay bars, saunas and clubs; an MSM mail-out condom programme; commercial venues (e.g. African food and barber shops); and in educational settings known to have large African student populations. Community organisations also made contact with potential participants through their regular online and face-to-face outreach and support work. Interviews took place in private spaces in partner organisations or in participants' own homes, and focused . The first part of the semi-structured interview focused on personal experiences of HIV, sexual health risk management practices, and use of sexual health technologies. The second part of the interview focused on the acceptability of PrEP and TasP, including . This included exploring awareness, potential use, concerns and combination with other and/or existing risk management strategies (such as TasP, serosorting, etc) (See Table 2).

PrEP was explained to participants by drawing on but not limited to the use of a visual aid (Figure 1). Basic explanations of PrEP were consistent across all FG and IDI discussions (Table 2, Section 4a). Subsequent and more detailed descriptions of PrEP varied depending on participant questions, which were encouraged and answered. This approach was taken to identify how PrEP should be described to potential candidates. Material did not specify an exact efficacy rate due to the emerging clinical data, variability according to adherence, and

For peer review only - http://bmjopen.bmj.com/site/about/guidelines.xhtml

to not overly complicate the explanation. Participants were informed that PrEP efficacy was dependent on levels of adherence, as demonstrated in a number of trials. FG participants were told that the iPrEx study reported approximately 73% protection if taken regularly (90% adherence), which was accurate at the time discussions were conducted (15). IDI participants were informed that efficacy could be up to or more than 90% if taken regularly, drawing on subsequent sub-analyses of clinical findings (2). Participants were informed that other forms of risk reduction were recommended, such as condoms (16) . Efficacy of condoms was described as less than 100% (17, 18)~~(18)~~. Discussions explored a wide-range of PrEP scenarios, including non-condom use.

Written consent was provided by all participants at the start of the FGs and IDIs. All FGs and IDIs were digitally recorded and transcribed verbatim. Transcripts were anonymised and coded in NVivo 10. Data were analysed thematically, drawing on both anticipated and emergent themes (19-21). Rigour throughout the analysis was achieved through an iterative process of discussion and revision between co-authors (19, 20, 22). ~~We have identified extracts taken from FGs; otherwise, it can be assumed that the extract comes from an IDI participant.~~ Ethical approval was obtained from the University of Glasgow's College of Social Sciences Ethics Committee (CSS2012/0193, CSS20120264).

Results

We identified five potential barriers to effective PrEP use: interpreting effectiveness; managing adherence; PrEP candidacy and low perceptions of HIV risk; concerns with other risks such as the criminalization of HIV transmission and STIs; and moral barriers. We have

For peer review only - http://bmjopen.bmj.com/site/about/guidelines.xhtml

identified extracts taken from FGs; otherwise, it can be assumed that the extract comes from an IDI participant.

Interpreting effectiveness

Understandings of PrEP effectiveness emerged as an important barrier to potential and effective use. Although participants were informed that PrEP was highly effective when taken regularly, most participants expressed concerns that it provided less than 100% protection and therefore was 'insufficient' to prevent HIV transmission on its own. Some participants felt that PrEP used in isolation was 'too much' of a risk (if only approximately 70% effective).

> ***Respondent 1****: That's not enough, that's not enough, exactly.*
>
> ***Respondent 2:*** *Its seventy-two per cent effective but then there's still that twenty-eight per cent.*
>
> (HIV-Negative MSM, FG)

Participants also expressed a wider scepticism of PrEP, which emerged in two ways. Some thought that the advice to use condoms with PrEP indicated a continued scientific uncertainty: '*I don't know if this [will] work because since they said if you use it you can still use condom, that means they are not sure as well'* (HIV-negative African woman). Second, some expressed scepticism in relation to the variability in reported efficacy rates in relation to adherence. As a result, there was a reluctance to trust these, unless the information came from a recognised, trusted and reliable source:

> ***R1:*** *There's so much out there right, on the internet an' everything else right, if it came up on the news right? The logistic news on BBC one that this pill will prevent*

For peer review only - http://bmjopen.bmj.com/site/about/guidelines.xhtml

so…—like a hundred per cent – that's when I would believe it would work. I [would not] trust any other pill …

Question: *So it has to be a hundred per cent?*

R2: *'cause there's a lot a... no like—anyone can put something on the internet,... You've got to use like certain websites, you've got to look like .org, .gov, those type o' websites. You've just done one that's like www.medicine.co.uk it's not a... it's not like a legitimate website it could just be anyone postin' stuff up there.*

Q: *Well I suppose in the States it's from the Food and Drug Administration so it's got that stamp on it.*

R1*: Yes, but you [don't know] if they're been a hundred per cent accurate on their job or just been lazy an' the fact that we're brave enough to take it to human trials right?*

(HIV-Negative MSM, FG)

In spite of being told that the FDA had approved the drug, these FG participants did not recognise the organisation or trust it as a reliable source, and were therefore sceptical about its statements and/or endorsement.

Other participants were less concerned about PrEP not being 100% efficacious because they imagined that it would be used *in addition* to condoms. However, participants expressed confusion or uncertainty in knowing how to interpret efficacy rates and their impact on risk reduction in this particular context. For example, in discussing the effectiveness of condoms in relation to other prevention strategies, one participant calculated the protection offered by PrEP, condoms and TasP with a hypothetical HIV-positive sexual partner. Assuming that PrEP was 90% effective at reducing HIV transmission, he explained:

For peer review only

R: but there's obviously still a 10% risk but, as you said, there's the same risk with condoms. So it's either you take the 10% risk or you say 'well, I'll use condoms and we'll use TASP' which makes 180%. (Laugh)

Q: And PrEP. (Laugh)

R: 253 out of 300!

(HIV-negative MSM)

Although the participant in this extract was joking about adding up the numbers, the interpretation of efficacy rates in relation to other prevention options posed a potential source of confusion or misinformation for participants.

Managing adherence

Given the evidence concerning the patterning of efficacy by adherence (2), maintaining regular adherence to medication was identified as a potential barrier to effective PrEP use. Some participants described how they might forget to take tablets or their routine might be disrupted because of non-regular working patterns, and were therefore concerned about how effective PrEP would be if they did not take the drugs regularly: '*Sometimes I forget. Because I don't have a regular sleeping pattern, so sometimes I'll fall asleep at four in the afternoon and I usually take my pills [for an existing health condition] after dinner'* (HIV-Negative MSM). Establishing and maintaining a routine to take PrEP was also affected by the perception of social stigma attached to HIV medication. This meant maintaining daily adherence would be difficult if there was limited privacy (e.g. the presence of room-mates) or if there was a change in environment. One participant reported concerns about his family potentially finding the tablets:

...if I was on those medications for a year and if I went home...or anything like that I'd find it very difficult to be able to take my medications or I would find it a bit of a

For peer review only

barrier that if my family knew about it they'd investigate why are you on these pills. And again that would probably put some doubt in their head and then they'd probably then think the worst - that I was HIV positive.

(HIV-negative MSM)

In addition to being unable to establish or maintain a regular routine to facilitate good adherence, most participants expressed concerns about the physical effects of the drugs themselves, and how the side-effects might inhibit taking them. Some participants described disliking taking tablets and that this would be a barrier to daily PrEP use: *I don't really like pills so I don't think I would take any pills every day'* (HIV-negative African man). Many viewed the potential for side effects, such as nausea and diarrhoea, as too great a trade-off for increased HIV prevention, in spite of being informed that trials had reported high tolerance of the drugs:

But, could I put up wi' side-effects all the time as well, like taking a pill, and going tae a nightclub, and ended up wi' diarrhoea? You know, you've gotta think of things like that, am I [going to] take this? Am I [going to] stand tired, have a headache in a club just at the fact that I might at the end of it get a shag...(HIV-Negative MSM)

Other participants felt the side-effects would interfere with sexual practice itself: '*Nausea, an' diarrhoea an'—you'll be shittin' all over his [penis]. No [thanks].'* (HIV-negative MSM, FG) A ~~significant~~ large minority of both HIV-positive and HIV-negative participants raised the issue of longer-term side-effects, especially those who were more familiar with the effects of ARVs:

Does it not have any side effects inside my body? 'Cause from my reading as well, some of these anti-retrovirals have got side-effects with the lungs, with the kidneys

For peer review only

and stuff. So, in the long run, if I get used to drinking this pill and I'm actually not exposed because I'm thinking I might be at risk of getting exposed, am I not doing more damage to myself in my body? (HIV-Negative African woman)

Many participants described scepticism in taking PrEP daily, especially if they were not always exposed to or at risk of HIV transmission.

PrEP candidacy and low perceptions of HIV risk

Many participants described scepticism in taking PrEP daily, especially if they were not always exposed to or at risk of HIV transmission. As such, Pperceptions of HIV transmission risk played an important role in potential uptake of PrEP. Several participants rejected the use of PrEP because they perceived themselves to be at a very low risk. For the majority of HIV-negative participants, this was because most could not imagine knowingly having an HIV-positive sexual partner adopted serosorting or believed they could accurately tell if a potential partner was HIV-positive and avoid sex with him or her: '*I wouldn't just [want to] take a risk because it's already defining as a risk, I mean, it's a risk, it's already been defined as a risk and since I'm a little bit risk averse... I won't just do anything'* (HIV-negative African man). HIV stigma and assumptions about disclosure appeared to inform these strategies. For example, Ssome HIV-negative participants assumed that HIV-disclosure to sexual partners '*was the law'* (HIV-negative MSM, FG), and that their sexual partners were not HIV-positive. Those HIV-negative participants in sero-discordant relationships and HIV-positive participants suggested that condoms, and the nature of their relationships (e.g. monogamous), already helped manage their risks, and that the additional benefit from PrEP was unnecessary. One man in a sero-discordant relationship explained:

For peer review only - http://bmjopen.bmj.com/site/about/guidelines.xhtml

Right now in my current situation as, I'm doing a monogamous relationship and everything like that, again I don't think even in that [instance] I don't think I would take the pills even though it would be an extra measure...I think I would feel comfortable enough in the current situation. (HIV-Negative MSM)

PrEP and concerns with other risks

Both HIV-positive and HIV-negative participants identified risk of other sexually-transmitted infections (STIs) as a concern for both themselves and their sexual partners. Heterosexual African participants, especially African women, also talked of the risk of pregnancy. For those participants who were either living with HIV, or who had experience of sero-discordant relationships, other health risks posed to the HIV-positive sexual partner through PrEP use were also identified:

when I'm looking at condom use it's not just HIV that you're protecting yourself against but some other STIs as well. So you're killing two birds with one stone, more like it. But when it's now using this, you're actually getting yourself involved in something that you already know – 'this person is positive and I'm [going to] have sex with him, but it's okay, I will not use condoms because I know I can use this.' But you're also putting that person, because that person's immune system might be weak. They're also putting that person at risk of other STIs as well, instead of using condoms. So I think it makes people more ignorant of the other things as well. And it makes people just more focused on just HIV and not other STIs. And then risk of pregnancy as well is likely to get high, provided the people are not on any other form of contraception.... No, I think... no, from the way you've said it to me... I wouldn't even tell it to people (HIV-negative African woman)

For peer review only

Most HIV-positive participants described anxieties about PrEP in relation to *their* inability to be in effective control of HIV prevention. One woman could not imagine agreeing to her HIV-negative sexual partner using PrEP: *'[PrEP] is too risky for him because I don't know when he stop using it, what will happen to him'* (HIV-positive African woman, FG).

Moral barriers to PrEP

PrEP emerged as a highly contentious issue because of the perceived negative implications it had for existing risk management strategies and HIV prevention. Many participants viewed PrEP as problematic because they perceived that *others* would stop using condoms if PrEP was available. That is, they distinguished between how *they* might use PrEP and how they perceived others would use it. For some, the concern was in relation to reduced condom use: '*it'll be like... women burning their bras. It'll be all these guys [whipping] off their condoms, do you know what I mean?'* (HIV-negative MSM). Other participants were also concerned with bigger changes in sexual practice, such as increased risk taking:

> *Are you trying to eliminate the condom?... There is no place for a condom at all when it's like this. So I think it actually encourages people to be more promisc... not promiscuous, but to be more... I don't know [if] 'ignorant' is the right word, but to be less careful 'cause they know 'oh, there's that pill'. And it's not even cost-effective than just getting a condom or using...PEP once in a blue moon.* (HIV-negative African woman)

Not all participants were opposed to PrEP and many viewed PrEP as a good addition to HIV prevention options. However, there was still concern about *how* PrEP would be accessed.

For peer review only - http://bmjopen.bmj.com/site/about/guidelines.xhtml

Overall it was hoped that it would be made available only under strict conditions to ensure the 'right' people received it and that it was used in the 'correct' way:

I believe it should be available in Scotland. But, it should be under strict control to make sure the person who takes it knows that he, or she is not protected to full extent from other infections, and HIV either. (HIV-negative MSM)

Discussion

This is the first qualitative study in the UK to report on the acceptability of PrEP. Our research offers new insights into the psychological and social barriers to PrEP uptake and use. We identified the significance of how the effectiveness of PrEP as a risk reduction intervention is communicated to and understood by potential candidates. ~~We demonstrated~~ Our research suggests the importance of HIV risk perception and found that, for many participants, PrEP was not immediately seen as a trusted and/or beneficial addition to their repertoire of existing risk reduction practices. Our findings also highlight how existing risk management strategies in relation to PrEP encompass broad concerns relating to sexual health, relationships, social factors and communities.

Understanding how to interpret PrEP efficacy rates, both on their own and in combination with other prevention strategies, proved a stumbling block for the participants and poses a considerable challenge to how health providers support the concept of combination prevention in the context of PrEP. Liu *et al* identify accurate consumer knowledge as key to PrEP implementation, in addition to addressing other factors such as stigma, adherence, and risk reduction (23). While we agree with Liu, our findings suggest that the form and delivery of this consumer knowledge, including *how* health providers understand and communicate

For peer review only - http://bmjopen.bmj.com/site/about/guidelines.xhtml

this information, needs further attention to support effective PrEP use. Communicating PrEP effectiveness in real world settings will be a two-way process that demands clarity on the part of both providers and potential users. In addition to supporting providers, ~~N~~negotiating PrEP as a prevention strategy~~, either on its own or with other strategies,~~ will require improved levels of HIV literacy amongst potential PrEP users to be effective. We suggest the need to consider *critical* HIV literacy ~~skills~~, which encompasses the ability to know, understand and use HIV-related information within existing risk reduction practices (24). As ~~Our findings suggest that~~ levels of HIV ~~literacy~~ knowledge are~~is~~ directly affected by a range of factors, such as ~~geography, migration status, and sero-status~~proximity to HIV(25), i ~~and that i~~nequalities in HIV literacy within communities affected by HIV will play an important role in understanding the barriers to PrEP use. These factors have direct implications for both the nature of how PrEP-related HIV risk prevention is delivered and by whom.

For many participants, PrEP was not seen as a necessary or welcome addition to their repertoire of risk management strategies. Our findings suggest that risk perception and candidacy will play a critical role in decisions to use PrEP, a finding echoed by Golub *et al* (26)~~(25)~~. For some of the HIV-negative and/or untested participants in our study, the rejection of PrEP as unnecessary emerged from a perception that they were not at risk of HIV transmission. HIV risk was managed often through the sexual exclusion of HIV-positive sexual partners or through reliance on monogamous sexual relationships. These findings suggest that PrEP implementation strategies will need to engage with these wider, socially embedded risk reduction practices, including how HIV stigma might affect risk perception. Moreover, our research ~~demonstrates~~ highlights how the management of risk was not limited only to HIV transmission, but also to risk of STIs, pregnancy and social stigma. Participants described how PrEP would not adequately address existing risks, and had the potential to

For peer review only

For peer review only - http://bmjopen.bmj.com/site/about/guidelines.xhtml

create significant new risks. Saberi *et al* reported similar moral concerns about the implications of PrEP on condom use in their study with MSM participants in serodiscordant relationships in the US (27)~~(26)~~. Although they surmise that proximity to HIV and age play a role in these concerns, our findings suggest that moral objections to PrEP were not limited by age or sexuality. We suggest that these moral reactions to PrEP as a risk reduction option are related to broader social and community concerns about the potential for PrEP to radically change the way prevention is practiced (9). This highlights the need to engage with wider social concerns about what constitutes 'inappropriate' or high-risk sexual practice in relation to PrEP and to demonstrate how PrEP implementation can be a part of a safe and comprehensive risk management strategy.

Our study has a number of strengths and limitations. We employed rigorous qualitative methodology which enabled in-depth exploration of the social meanings of PrEP acceptability and likely use (21). We therefore add to existing quantitative PrEP acceptability research. With a small sample of MSM and migrant African participants in a non-generalized HIV epidemic~~.~~, some of whom were engaged in sexual health or community services, our findings are not generalisable to a wider population but we would argue, are transferable to similar populations in similar social contexts. As we did not sample according to sexual risk behaviour, our findings are only transferrable to broad risk groups and not necessarily to 'high risk' individuals. Our inclusion of the recommendation to use condoms in the visual PrEP information may have biased the findings. However, our discussions included consistent and broad descriptions of PrEP and encompassed a wide range of PrEP scenarios, including non-condom use.

For peer review only

For peer review only - http://bmjopen.bmj.com/site/about/guidelines.xhtml

Our research ~~demonstrates~~ identifies the need to consider acceptability factors which extend beyond drug adherence and risk compensation when introducing and scaling up PrEP and has a number of implications for policy and clinical practice. In particular, it will be necessary to develop clear tools and techniques to communicate PrEP information to potential candidates, and to support health providers in the implementation of these tools. These methods will need to translate clinical research relating to PrEP effectiveness in real world contexts as is appropriate in the context of diverse critical literacy skills. Implementation will also need to address low risk perception, non-HIV related risk reduction and other moral concerns to demonstrate how PrEP can be a part of a safe and comprehensive risk management strategy. Our study suggests that broader social and community concerns about the potential for PrEP to change HIV prevention need to be addressed by supporting the integration of PrEP into existing risk management strategies and through targeted promotion.

Competing Interests

We have no competing interests.

Author's Contributions

IY designed the study, carried out the qualitative data collection and analysis and drafted the manuscript. PF helped to design the study, carried out some of the data collection and analysis and helped to draft the manuscript. LMD participated in the design of the study and helped to draft the manuscript. All authors read and approved the final manuscript.

Acknowledgements

The HIV and the Biomedical Study, IY and LMD are core funded by the UK Medical Research Council (MRC) (MC_U130031238/MC_UU_12017/2) at the MRC/Chief Scientist Office (CSO) Social and Public Health Sciences Unit, University of Glasgow. PF is funded by Glasgow Caledonian University. We thank the organisations (Waverly Care, Terrence

For peer review only - http://bmjopen.bmj.com/site/about/guidelines.xhtml

Higgins Trust Scotland, LGBT Youth Scotland, Gay Men's Health Scotland, Positive Scotland, NHS Greater Glasgow and Clyde, NHS Lothian, NHS Grampian) who helped with recruitment and the men and women who agreed to take part in the focus groups and interviews.

References

1. Baeten JM, Grant R. Use of Antiretrovirals for HIV Prevention: What Do We Know and What Don't We Know? Current HIV/AIDS Reports. 2013;10(2):142-51.

2. Anderson PL, Glidden DV, Liu A, Buchbinder S, Lama JR, Guanira JV, et al. Emtricitabine-Tenofovir Concentrations and Pre-Exposure Prophylaxis Efficacy in Men Who Have Sex with Men. Science Translational Medicine. 2012;4(151):1-8.

3. Young I, McDaid L. How Acceptable are Antiretrovirals for the Prevention of Sexually Transmitted HIV?: A Review of Research on the Acceptability of Oral Pre-exposure Prophylaxis and Treatment as Prevention. AIDS Behav. 2014 Jul 30;18 (2):195-216.

4. Holt M. HIV pre-exposure prophylaxis and treatment as prevention: a review of awareness and acceptability among men who have sex with men in the Asia-Pacific region and the Americas. Sex Health. 2013 Jul 19.

5. Gamarel KE, Golub SA. Intimacy Motivations and Pre-exposure Prophylaxis (PrEP) Adoption Intentions Among HIV-Negative Men Who Have Sex with Men (MSM) in Romantic Relationships. Annals of Behavioral Medicine. 2014:1-10.

6. Kirby T, Thornber-Dunwell M. Uptake of PrEP for HIV slow among MSM. Lancet. 2014;383(9915):399-400.

7. Amico KR, Stirratt MJ. Adherence to Preexposure Prophylaxis: Current, Emerging, and Anticipated Bases of Evidence. Clinical Infectious Diseases. 2014 July 1, 2014;59(suppl 1):S55-S60.

For peer review only

8. Cairns G. The road to PrEP: trials, regulation and roll-out. aidsmap. 2012.

9. Belluz J. The Truvada wars. BMJ. 2014(348):g3811

10. Kippax S, Stephenson N. Beyond the distinction between biomedical and social dimensions of HIV prevention through the lens of a social public health. Am J Public Health. 2012 May;102(5):789-99.

11. Nguyen VK, Bajos N, Dubois-Arber F, O'Malley J, Pirkle CM. Remedicalizing an epidemic: from HIV treatment as prevention to HIV treatment is prevention. AIDS. 2011 Jan 28;25(3):291-3.

12. Health Protection Scotland. ANSWER: HIV infections and AIDS in Scotland: Quarterly report to 30 June 2013: Health Protection Scotland2013 4 September 2013 Contract No.: 2013/36.

13. PHE. HIV in the United Kingdom: 2013 Report. London: Public Health England2013.

14. Ware NC, Wyatt MA, Haberer JE, Baeten JM, Kintu A, Psaros C, et al. What's love got to do with it? Explaining adherence to oral antiretroviral pre-exposure prophylaxis for HIV-serodiscordant couples. J Acquir Immune Defic Syndr. 2012 Apr 15;59(5):463-8.

15. Grant RM, Lama JR, Anderson PL, McMahan V, Liu AY, Vargas L, et al. Preexposure chemoprophylaxis for HIV prevention in men who have sex with men. New England Journal of Medicine. 2010;363(27):2587-99.

16. Center for Disease Control (CDC). Interim Guidance: Pre-exposure Prophylaxis for the Prevention of HIV Infection in Men Who Have Sex with Men. Morbidity and Mortality Weekly Report. 2011;60(3):65-8.

17. Cairns G. Consistent condom use in anal sex stops 70% of HIV infections, study finds. 4 March 2013 [10 September 2014]; Available from: http://www.aidsmap.com/Consistent-condom-use-in-anal-sex-stops-70-of-HIV-infections-study-finds/page/2586976/.

Field Code Changed

Formatted: Hyperlink, Font: (Default) +Body (Calibri), 11 pt

For peer review only - http://bmjopen.bmj.com/site/about/guidelines.xhtml

18. Weller S, Davis K. Condom effectiveness in reducing heterosexual HIV transmission. Cochrane Database Syst Rev. 2002;1(1).

19. Mason J. Qualitative Researching. London: Sage Publications; 1996.

20. Barbour R, Kitzinger J, editors. Developing Focus Group Research: Politics, Theory and Practice. London: Sage Publications; 1999.

21. Ziebland S, McPherson A. Making sense of qualitative data analysis: an introduction with illustrations from DIPEx (personal experiences of health and illness). Medical Education. 2006;40(5):405-14.

22. Silverman D. Doing Qualitative Research: A Practical Handbook. London: Sage; 2000.

23. Liu A, Cohen S, Follansbee S, Cohan D, Weber S, Sachdev D, et al. Early Experiences Implementing Pre-exposure Prophylaxis (PrEP) for HIV Prevention in San Francisco. PLoS Med. 2014;11(3):e1001613.

24. Sørensen K, Van den Broucke S, Fullam J, Doyle G, Pelikan J, Slonska Z, et al. Health literacy and public health: a systematic review and integration of definitions and models. BMC Public Health. 2012;12(1):80.

25. Keogh P. Morality, responsibility and risk: Negative gay men's perceived proximity to HIV. AIDS Care. 2008;20(5):576-81.

26. Golub SA, Gamarel KE, Rendina HJ, Surace A, Lelutiu-Weinberger CL. From efficacy to effectiveness: facilitators and barriers to PrEP acceptability and motivations for adherence among MSM and transgender women in New York City. AIDS patient care and STDs. 2013;27(4):248-54.

27. Saberi P, Gamarel KE, Neilands TB, Comfort M, Sheon N, Darbes LA, et al. Ambiguity, ambivalence, and apprehensions of taking HIV-1 pre-exposure prophylaxis

For peer review only - http://bmjopen.bmj.com/site/about/guidelines.xhtml

among male couples in San Francisco: a mixed methods study. PLoS One. 2012;7(11):e50061.

1. Baeten JM, Grant R. Use of Antiretrovirals for HIV Prevention: What Do We Know and What Don't We Know? Current HIV/AIDS Reports. 2013;10(2):142-51.

2. Anderson PL, Glidden DV, Liu A, Buchbinder S, Lama JR, Guanira JV, et al. Emtricitabine-Tenofovir Concentrations and Pre-Exposure Prophylaxis Efficacy in Men Who Have Sex with Men. Science Translational Medicine. 2012;4(151):1-8.

3. Young I, McDaid L. How Acceptable are Antiretrovirals for the Prevention of Sexually Transmitted HIV?: A Review of Research on the Acceptability of Oral Pre-exposure Prophylaxis and Treatment as Prevention. AIDS Behav. 2014 Jul 30;18 (2):195-216.

4. Holt M. HIV pre-exposure prophylaxis and treatment as prevention: a review of awareness and acceptability among men who have sex with men in the Asia-Pacific region and the Americas. Sex Health. 2013 Jul 19.

5. Gamarel KE, Golub SA. Intimacy Motivations and Pre-exposure Prophylaxis (PrEP) Adoption Intentions Among HIV-Negative Men Who Have Sex with Men (MSM) in Romantic Relationships. Annals of Behavioral Medicine. 2014:1-10.

6. Kirby T, Thornber-Dunwell M. Uptake of PrEP for HIV slow among MSM. Lancet. 2014;383(9915):399-400.

7. Amico KR, Stirratt MJ. Adherence to Preexposure Prophylaxis: Current, Emerging, and Anticipated Bases of Evidence. Clinical Infectious Diseases. 2014 July 1, 2014;59(suppl 1):S55-S60.

8. Cairns G. The road to PrEP: trials, regulation and roll-out. aidsmap. 2012.

9. Belluz J. The Truvada wars. BMJ. 2014(348):g3811

For peer review only - http://bmjopen.bmj.com/site/about/guidelines.xhtml

10. Kippax S, Stephenson N. Beyond the distinction between biomedical and social dimensions of HIV prevention through the lens of a social public health. Am J Public Health. 2012 May;102(5):789-99.

11. Nguyen VK, Bajos N, Dubois-Arber F, O'Malley J, Pirkle CM. Remedicalizing an epidemic: from HIV treatment as prevention to HIV treatment is prevention. AIDS. 2011 Jan 28;25(3):291-3.

12. Health Protection Scotland. ANSWER: HIV infections and AIDS in Scotland: Quarterly report to 30 June 2013: Health Protection Scotland2013 4 September 2013 Contract No.: 2013/36.

13. PHE. HIV in the United Kingdom: 2013 Report. London: Public Health England2013.

14. Ware NC, Wyatt MA, Haberer JE, Baeten JM, Kintu A, Psaros C, et al. What's love got to do with it? Explaining adherence to oral antiretroviral pre-exposure prophylaxis for HIV-serodiscordant couples. J Acquir Immune Defic Syndr. 2012 Apr 15;59(5):463-8.

15. Grant RM, Lama JR, Anderson PL, McMahan V, Liu AY, Vargas L, et al. Preexposure chemoprophylaxis for HIV prevention in men who have sex with men. New England Journal of Medicine. 2010;363(27):2587-99.

16. Center for Disease Control (CDC). Interim Guidance: Pre-exposure Prophylaxis for the Prevention of HIV Infection in Men Who Have Sex with Men. Morbidity and Mortality Weekly Report. 2011;60(3):65-8.

17. Cairns G. Consistent condom use in anal sex stops 70% of HIV infections, study finds. 4 March 2013 [10 September 2014]; Available from: http://www.aidsmap.com/Consistent-condom-use-in-anal-sex-stops-70-of-HIV-infections-study-finds/page/2586976/.

18. Weller S, Davis K. Condom effectiveness in reducing heterosexual HIV transmission. Cochrane Database Syst Rev. 2002;1(1).

Field Code Changed

For peer review only

For peer review only - http://bmjopen.bmj.com/site/about/guidelines.xhtml

19. Mason J. Qualitative Researching. London: Sage Publications; 1996.

20. Barbour R, Kitzinger J, editors. Developing Focus Group Research: Politics, Theory and Practice. London: Sage Publications; 1999.

21. Ziebland S, McPherson A. Making sense of qualitative data analysis: an introduction with illustrations from DIPEx (personal experiences of health and illness). Medical Education. 2006;40(5):405-14.

22. Silverman D. Doing Qualitative Research: A Practical Handbook. London: Sage; 2000.

23. Liu A, Cohen S, Follansbee S, Cohan D, Weber S, Sachdev D, et al. Early Experiences Implementing Pre-exposure Prophylaxis (PrEP) for HIV Prevention in San Francisco. PLoS Med. 2014;11(3):e1001613.

24. Sørensen K, Van den Broucke S, Fullam J, Doyle G, Pelikan J, Slonska Z, et al. Health literacy and public health: a systematic review and integration of definitions and models. BMC Public Health. 2012;12(1):80.

25. Golub SA, Gamarel KE, Rendina HJ, Surace A, Lelutiu-Weinberger CL. From efficacy to effectiveness: facilitators and barriers to PrEP acceptability and motivations for adherence among MSM and transgender women in New York City. AIDS patient care and STDs. 2013;27(4):248-54.

26. Saberi P, Gamarel KE, Neilands TB, Comfort M, Sheon N, Darbes LA, et al. Ambiguity, ambivalence, and apprehensions of taking HIV-1 pre-exposure prophylaxis among male couples in San Francisco: a mixed methods study. PLoS One. 2012;7(11):e50061.

For peer review only

For peer review only - http://bmjopen.bmj.com/site/about/guidelines.xhtml

Table 1: Focus Group Discussion Guide

Part 1

Participants were presented with a number of objects to discuss. Objects included: condoms, sachets of lubricant, pregnancy test, aidsmap list of ARVs, mocked up bottle of antibiotics, empty boxes/bottles of Truvada, and pictures of: Orasure quicktest and rapid HIV test

- **What do these objects make you think about?**
  - i.e. engagement with sexual health organisations? Own HIV status? Sexual encounters? Pleasure? Community? Privacy?
- **How do these objects relate to risk and HIV?**
  - Who uses these objects? Do you use these objects? Why, or why not?
- **What is risky in relation to HIV?**
  - Is it the person, the sexual position, the location, your own health?
- **Do you use these objects to manage HIV?**
  - If yes, how? If not, why not?
  - Does using these objects hinder/help management of HIV/disclosure of status to others?
- **What else do you use to manage HIV?**
  - i.e. how do you assess risk and how do you manage it? Gaydar profile? Looks?

Part 2

Provide visual cards of PrEP & TasP provided & explain separately.

- **How might you use these pills?**
  - Ask about serodiscordant partnerships and how these pills might change how HIV transmission is managed?
  - Would the potential use of these pills change depending on: sexual partner, current health experiences, location (space, place), etc?
- **How do you think your friends or sexual partners might use these pills?**
  - Would people choose these pills? Would they choose them in combination with condoms? Would they trust these pills would work? Would this depend on the sexual partner?
  - How would you decide to use them? How do you decide on current sexual health practices?
- **Are you concerned about the use of pills as a form of HIV prevention?**

For peer review only - http://bmjopen.bmj.com/site/about/guidelines.xhtml

- ~~Concerns about other STIs/Hep C?~~
- ~~Would the side effects be a problem? What are your current experiences with side effects and do you think other men could manage these?~~

- **Would these pills change the way people currently manage HIV? What do you think about this?**
    - ~~Ask about understandings/experiences of condom use, feelings of security/management of risk with existing options?~~
    - ~~Would this increase/decrease HIV related stigma?~~
    - ~~Would this change where the onus of responsibility was placed within the community? (i.e. are HIV positive people more or less responsible to manage transmission now, and would these pills/interventions change things?)~~

For peer review only - http://bmjopen.bmj.com/site/about/guidelines.xhtml

Table 2: Interview Topic Guide for HIV-negative or Untested Participants

1) **Experiences with and/or /proximity to HIV**

Before I start asking you questions specifically about the latest developments, I'd like to learn a little more about your own experiences with HIV. Can you tell me about that?

Confirm HIV Status: HIV negative/unknown status/undisclosed/other

- Is HIV a risk for you?
  - If yes, what are you concerned about in particular?
  - If you are not concerned about HIV, why not?
  - Has this changed at all for you? (different sexual partners, location, sexual practice, etc)
- Is HIV something that you talk about with your sexual partners? (or friends?)
  - If yes, what do you speak about? In what contexts would you talk about HIV?
  - If not, would you feel able to discuss HIV or any concerns you had about HIV with your sexual partners and/or friends?
- Have you ever tested for HIV?
  - If yes:
    - What prompted you get a test?
    - Where did you get the test done?
    - Have you tested for HIV more than once? How often do you get tested?
  - If not:
    - Have you thought about getting tested before?
    - Is there anything that's stopped you from seeking an HIV test?
    - What might make you get tested?

2) **Risk management/sexual health**

This study is about understanding how people manage their sexual health and risk of HIV.

- What are the risks for you in relation to sexual health?
- You've said that you manage risk in sexual health by ..... Has this changed and how?;
  - Has this changed over time?
  - Does this change according to sexual partner?
  - Does this change according to location? (home, travelling, public spaces)
- Do you talk to your sexual partners about thishow you manage your sexual health?
  - Why/Why not?
- Do you use health or other services to help you manage your sexual health? If so, how?

3) **Use of existing technologies** –

For peer review only - http://bmjopen.bmj.com/site/about/guidelines.xhtml

I'm interested in how people use existing sexual health technologies. This can include lots of things. Here is a list of some of the things that people might use.

List of sexual health technologies physically presented to participant:

condoms
HIV testing
Sexually transmitted infections (STI) testing
Contraception (the pill, IUD, long-term injections, etc)
pregnancy testing
CD4 counts
viral loads
Anti-retrovirals (ARVs)
post-exposure prophylaxis (PEP)

- Do you use any of these now? Have you used any of these in the past?
  - If not, is there anything that is missing from this list that you or people you know have used?
- How have you used them? What made you use them?
  - How do they work? (explore how is this related to understandings of risk of HIV transmission)
- Have you used any of these in combination with other prevention methods?
- How do you feel about using them?
  - Ease/difficulty of using these?
  - Social concerns?
  - Trust in effectiveness?
- If you started/stopped using some of these, can you say why you did?

4) Potential use of new technologies

a) PrEP

This study is about exploring how people feel about using ARVs, the medication currently used to treat HIV, as a way of preventing HIV. This can be done in a couple of ways.

Approximate PrEP description explained to participant:

Pre-exposure Prophylaxis (PrEP) is when ARVs are used by people who are HIV negative to prevent the transmission of HIV. At the moment, it can be taken once a day, although researchers are looking into other forms (short term PrEP, long-acting injectable, topical gel/microbicides). PrEP only works if people take the medication regularly. Clinical trials have shown that it is effective in relation to how often people take the pills. People are still encouraged to use condoms and other forms of risk reduction with PrEP use. There are some side effects, but this may not affect everyone and it generally seems to be well tolerated in the clinical trials. PrEP is not currently available in the UK, but it has been licensed for use in the United States.

Provide card, but verbally explain:

For peer review only

PrEP Pre-exposure Prophylaxis (PrEP) is when ARVs are used by people who are HIV negative to prevent the transmission of HIV.
At the moment, it can be taken once a day, although researchers are looking into other forms (short term PrEP, long-acting injectable, topical gel/microbicides).
PrEP only works if people take the medication regularly. Clinical trials have shown that it is effective in relation to how often people take the pills.
People are still encouraged to use condoms and other forms of risk reduction with PrEP use.
There are some side effects, but this may not affect everyone and it generally seems to be well tolerated in the clinical trials.
PrEP is not currently available in the UK, but it has been licensed for use in the United States.

- Have you heard of PrEP before?
  - If so, where? From whom? What had you heard?
- What do you think of PrEP as a prevention method?
- How would you feel about using PrEP as a prevention method?
  - If would not, why not?
  - If good, why?
- If you would use PrEP, how do you think you would you use it?
  - In combination with other forms of prevention? As a back-up?
  - In what circumstances might you see PrEP as a potential prevention method?
- Do you have any concerns about PrEP as a prevention method?
  - Concerns about taking medication to prevent HIV transmission? (interactions, remembering tablets, side effects, etc)
  - Do you have any concerns about how it might work?
  - Would there be any social stigma associated with PrEP?
- Do you think other people might use PrEP as a method?
  - What do you think about that?

b) TasP

Approximate TasP description explained to participant:

Treatment as prevention is when ARVs are used by people living with HIV to not only to clinically manage HIV, but also to help prevent the transmission of HIV. TasP manages the 'viral load' or the amount of HIV in the system. Research has show that having an 'undetectable' viral load means that transmission of HIV is unlikely to happen. So, if the HIV positive person is taking their treatment regularly, and they don't have an STI, and their viral load is 'undetectable' for a period of time (about 6 months), they would clinically be considered not infectious. TasP in particular is when treatment is started to prevent transmission, rather than when the person clinically needs the treatment. If someone starts this treatment early – including for prevention reasons – they cannot stop taking this medication. So although people living with HIV will eventually move onto treatment, perhaps after 5 to even 10 years, this would mean starting treatment considerably earlier.

TasP

Provide card, but verbally explain:

Treatment as prevention is when ARVs are used by people living with HIV to not only to clinically manage HIV, but also to help prevent the transmission of HIV.
TasP manages the 'viral load' or the amount of HIV in the system. Research has show that having an 'undetectable' viral load means that transmission of HIV is unlikely to happen. So,

For peer review only - http://bmjopen.bmj.com/site/about/guidelines.xhtml

~~if the HIV positive person is taking their treatment regularly, and they don't have an STI, and their viral load is 'undetectable' for a period of time (about 6 months), they would clinically be considered not infectious.~~
~~TasP in particular is when treatment is started to prevent transmission, rather than when the person clinically needs the treatment.~~
~~If someone starts this treatment early – including for prevention reasons – they cannot stop taking this medication. So although people living with HIV will eventually move onto treatment, perhaps after 5 to even 10 years, this would mean starting treatment considerably earlier.~~

- Have you heard of TasP before?
  - ~~If so, where? From whom? What had you heard?~~
- What do you think of TasP as a prevention method?
  - ~~What do you think about using drugs to lower levels of infection in people living with HIV?~~
- Can you imagine using this as a prevention method with a sexual partner who is HIV positive?
  - ~~If yes, why?~~
  - ~~If not, why not?~~
- How would you feel if a sexual partner suggested this as an HIV prevention method?
- Do you have any concerns about this as a prevention method?
  - ~~Trust in viral load levels/science?~~
  - ~~Concerns with social perceptions? (i.e. social stigma around ARVs)~~
  - ~~Coercion/pressure by sexual partners not to use condoms?~~
  - ~~Anxieties around STIs?~~
- How do you think other people who are HIV negative or untested might feel about using ARVs or HIV treatment as a prevention method?
  - ~~What do you think about that?~~

~~**General themes**~~
~~In addition to the above themes/areas of enquiry, the interview will pay particular attention to cross-cutting themes and issues such as:~~
- ~~Gender and sexuality~~
- ~~Social stigma (HIV-related or otherwise)~~
- ~~HIV & sexual health literacy~~
- ~~HIV serostatus as an identity (or not) and how this might function/influence social practices~~
- ~~Community norms and practices in relation to sexual relations and sexual health~~
- ~~Social and structural barriers/enablers to sexual health & use of technologies (i.e. access to services, cost, stigma, understandings of rights, legal issues, etc)~~
- ~~Individual vs group rights and responsibilities~~
- ~~Trust in science, trust in people~~

For peer review only - http://bmjopen.bmj.com/site/about/guidelines.xhtml

Figure 1.

PrEP (Pre-Exposure Prophylaxis)

**Who is it for?**	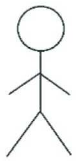 **Person not living with HIV (HIV negative)**
**What is it?**	ARVs are taken **before exposure to HIV** (usually before having sex with someone living with HIV)
**What does this involve?**	PrEP would mostly likely be a pill that would be taken everyday. Side effects could include nausea, diarrhoea, headaches, and tiredness.
**Does this work?**	PrEP is not 100% effective, but **when used with condoms, significantly reduces risk of HIV transmission**.
**Can I get it here?**	No, PrEP is not currently available in Scotland or anywhere else in the UK.

160x225mm (300 x 300 DPI)
